# Black Sigatoka in bananas: Ecoclimatic suitability and disease pressure assessments

**DOI:** 10.1371/journal.pone.0220601

**Published:** 2019-08-14

**Authors:** Tania Yonow, Julian Ramirez-Villegas, Catherine Abadie, Ross E. Darnell, Noboru Ota, Darren J. Kriticos

**Affiliations:** 1 HarvestChoice, InSTePP, University of Minnesota, St. Paul, MN, United States of America; 2 CSIRO, Canberra ACT, Australia; 3 International Center for Tropical Agriculture (CIAT), Cali, Colombia; 4 CGIAR Research Program on Climate Change, Agriculture and Food Security (CCAFS), Cali, Colombia; 5 Visiting Research Fellow, School of Earth and Environment, University of Leeds, Leeds, United Kingdom; 6 BGPI, Univ Montpellier, CIRAD, INRA, Montpellier SupAgro, Montpellier, France; 7 CIRAD, UMR BGPI, F-97130 Capesterre Belle-Eau, Guadeloupe, France; 8 CSIRO, Brisbane, QLD, Australia; 9 The University of Queensland, School of Biological Sciences, St. Lucia, QLD, Australia; University of Thessaly School of Agricultural Sciences, GREECE

## Abstract

Black leaf streak disease, or black Sigatoka, is caused by the fungus *Pseudocercospora fijiensis*, and has been identified as a major constraint to global production of banana and plantain. We fitted a climatic niche model (CLIMEX) for *P*. *fijiensis* to gain an understanding of the patterns of climate suitability, and hence hazard from this disease. We then calibrated the climate suitability patterns against the results of an expert elicitation of disease pressure patterns. We found a moderately strong non-linear relationship between modelled climate suitability for *P*.*°fijiensis* and the expert ratings for disease pressure. The strength of the relationship provides a cross-validation between the CLIMEX model and the expert elicitation process. The bulk of global banana production experiences high potential threat from *P*. *fijiensis*, and the higher yielding areas for banana and plantain production are at greatest threat. By explicitly considering the role of irrigation we have been able to identify how strategic irrigation could be used to support banana production in areas that are at low risk from *P*. *fijiensis*.

## Introduction

Black leaf streak disease (BLSD), also known as black Sigatoka disease, has long been a major constraint to banana and plantain production [1–5], inducing severe defoliation, and reducing photosynthesis, biomass accumulation and yield across most of the tropical and sub-tropical environments where bananas and plantains are cultivated. It is one of the most serious biological threats to banana production for food security and export [6–8]. Control of BLSD has been increasing over the years [9], originally accounting for 27% of production costs in Central America [1] and yield losses in excess of 38% [10]. The primary impact of BLSD on export production is a decrease in the greenlife of harvested fruits [10, 11]. Some countries have ceased exporting bananas due to the high costs of producing fruit of export quality [5]. Only a few countries that grow banana plants commercially are free of this disease: India, Pakistan, South Africa, Israel and mainland Australia [12]. BLSD is caused by the ascomycete fungus *Pseudocercospora fijiensis* (M. Morelet) Deighton, formerly known as *Mycosphaerella fijiensis* M. Morelet.

*Pseudocercospora fijiensis* originated in Southeast Asia [5, 6], with the oldest record of *P*. *fijiensis* being from southern Taiwan in 1927 [13]. Southeast Asia is also the origin of the host plant [14]. In Asia, *P*. *fijiensis* is now present in Bhutan, China, Indonesia (including Java and Sumatra), Malaysia, the Philippines, Singapore, Taiwan, Thailand and Vietnam. It occurs on many islands of Australasia and Oceania: American Samoa, Torres Strait Islands of Australia, Cook Islands, Fiji, French Polynesia (Tahiti), Mariana Islands, Micronesia, New Caledonia, Niue, Norfolk Island, Papua New Guinea, Samoa, Solomon Islands, Tonga, Vanuatu, Wallis and Futuna Islands. Although the “… exact distribution of black Sigatoka in Asia is not known” [15], it is likely to be more widespread than has been reported.

*Pseudocercospora fijiensis* can disperse naturally over short distances (within plots and farms), [16], and anthropogenically over long-distances, via movements of infested plants between countries or continents [14]. It was introduced to Central America in Honduras in 1972 after independent and multiple introductions from Oceania and South East Asia [17]. From Honduras, the disease spread to the rest of Central America and into South America [17], North America [5], and the Caribbean [8]. In Africa, *P*. *fijiensis* was first reported in Gabon in 1978 [18], and has since spread to West Africa and East Africa [19]. It has been suggested that African *P*. *fijiensis* came from a single introduction event from Asia [14].

The spread of *P*. *fijiensis* from its origin has been summarised elsewhere [5, 6, 15, 20] and its distribution presented as maps [21, 22]. Despite the importance of *P*. *fijiensis* as a constraint to banana and plantain cultivation, existing maps lack the level of detail necessary for spatial targeting of BLSD management, or for predicting the potential spread of *P*. *fijiensis* from neighbouring areas. More detailed knowledge of the geographical distribution of *P*. *fijiensis* and its climate and management drivers is needed to better manage BLSD, and to control its dispersal into new areas.

The aim of this study is to map the potential distribution of *P*. *fijiensis* as determined by climate and modified by irrigation. To do this, we use CLIMEX (version 4), a process-oriented niche model that describes species’ responses to climatic variables [23, 24]. We selected CLIMEX for the analysis because it has been shown to be superior to other modelling frameworks for estimating the potential geographical ranges of species [25, 26]. We parameterise the CLIMEX model using 137 presence location records from 25 countries, and validate it with an independent dataset of 119 presence locations from 48 countries. We use the fitted parameters to map the global distribution of *P*. *fijiensis* as determined by climate as well as the associated model uncertainty. Because the use of certain irrigation systems may increase disease severity [27], we explicitly model and map the role of irrigation in modifying habitat suitability and in making otherwise inclement environments suitable for the persistence and growth of *P*. *fijiensis*. We also test whether the fitted climate suitability model can act as a reliable index of expert rankings of disease pressure (6 experts for 88 localities in 20 countries), and finally, we consider the spatial relationship between disease pressure and production of banana and plantain.

## Methods

We use the CLIMEX process-based niche model to map the climate- and management-controlled distribution of *P*. *fijiensis*. CLIMEX uses an Ecoclimatic Index (EI) to describe the suitability of locations for population growth and survival. We parameterise the CLIMEX model using 137 presence location records from 25 countries, and validate the fitted model with an independent dataset of 119 presence locations from 48 countries. We further test whether the fitted climate suitability model can act as a reliable index of expert rankings of disease pressure (6 experts for 88 localities across 20 countries). We then use the fitted parameters to map the global potential distribution of *P*. *fijiensis*, as well as the associated prediction uncertainty. In assessing the management-climate-controlled distribution, we explicitly model the role of irrigation in modifying habitat suitability, and in making otherwise inclement environments suitable for the persistence and growth of *P*. *fijiensis*. In the following sub-sections we describe all data and methods used to conduct our analyses.

### Disease occurrence records

Geocoded location records were collated from three sources. The first source was a global expert-based dataset of disease pressure (disease severity) covering 88 localities from 20 countries (S1 Fig). BLSD expert researchers were asked to pinpoint locations in Google Earth where they had conducted field work and/or had performed field visits, and they knew *P*.*°fijiensis* was present. This dataset was initially used for model fitting purposes, and subsequently used in an analysis examining the relationship between the model output and disease pressure/severity.

An additional 49 localities from 11 countries (five of which were not in the expert dataset) were sourced from published literature (S1 Fig). In some cases, this included records with town name localities. We recognise that although agriculture is not done in towns *per se*, these records should nonetheless be representative of where the disease was recorded. This dataset contributed to the total pool (137 location records from 25 countries) of geocoded location records used to fit the model.

The third source of geocoded location records was used to validate the final model. This dataset included 119 locations from 48 countries: 33 locations from 14 countries in Asia, 22 locations from 13 countries in Africa, and 64 locations from 21 countries in the Americas and the Caribbean (S1 Fig). The presence of BLSD at these locations was confirmed by isolation of *P*. *fijiensis* for genetic studies [14].

### Meteorological data

For all model runs, we used the CM10_1975H V1.1CliMond climatic dataset [28]. This dataset comprises 30-year averages of monthly values for daily minimum and maximum temperature (°C), relative humidity (%) at 09:00 and 15:00, and monthly rainfall total (mm).

### Mapping

**Fig 1:**
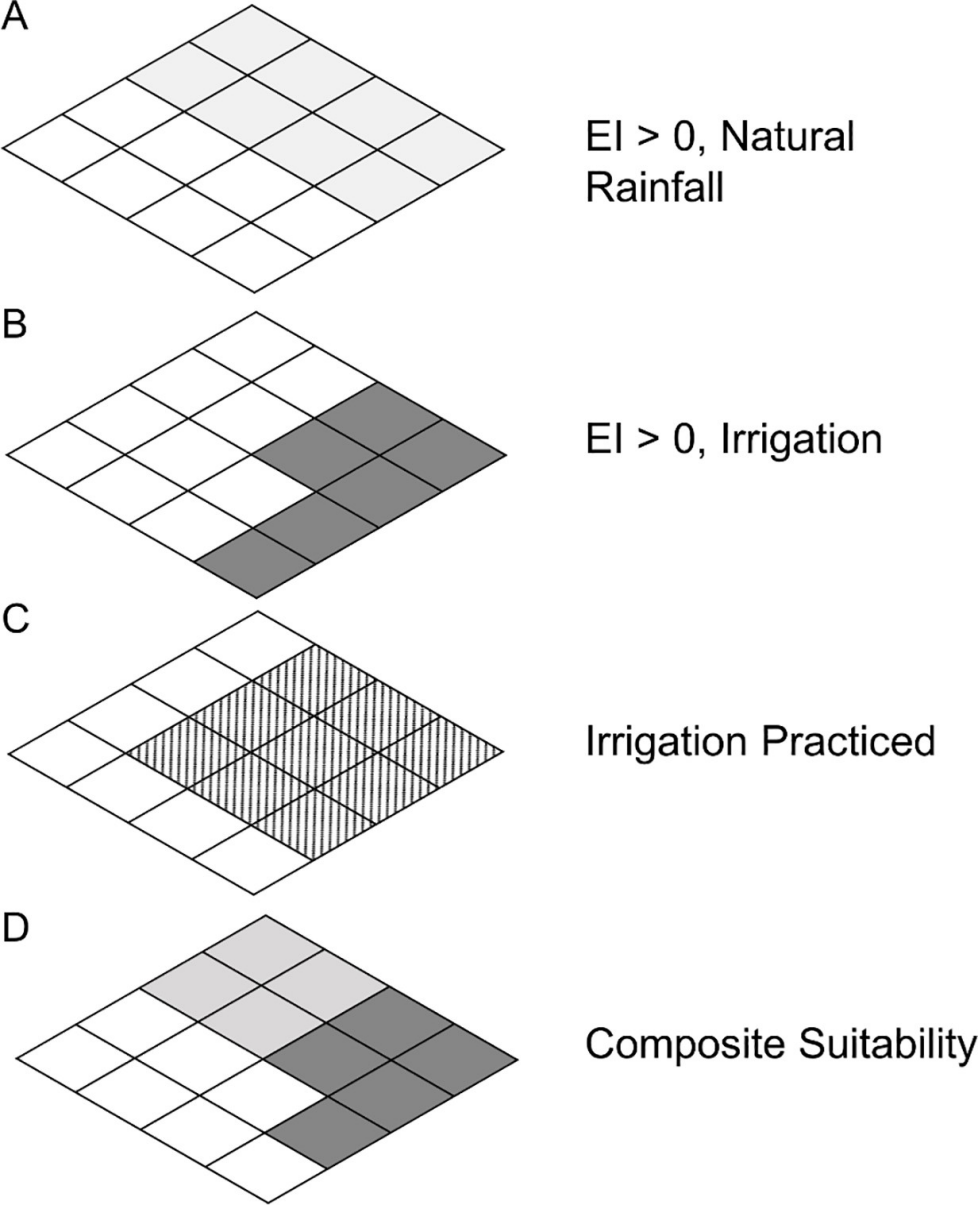
Mapping composite climate suitability. (A) Shaded areas are suitable under a natural rainfall scenario; (B) shaded areas are suitable under an irrigation scenario; (C) hatched areas are identified irrigation areas [33]; (D) composite climate suitability is calculated as the maximum EI values of shaded areas in (A), and shaded areas in (B) where irrigation is practiced [hatched areas in (C)].

As in previous work where irrigation is explicitly considered as having a mitigating influence on the distribution of a species [29–32], we provide maps of the projected distribution of *P*. *fijiensis* under a natural rainfall scenario and as a composite of both the natural rainfall scenario and the 5 mm day^-1^rainfall scenario. The composite climate suitability maps use identified irrigation areas [33]. For each 10' cell, if the irrigated area was greater than 2.5ha, the irrigation scenario result was used. Otherwise, the natural rainfall scenario result was used (Fig 1).

### Model parameterisation

We used the CLIMEX model [23, 24] to map the distribution of *P*. *fijiensis* globally. CLIMEX is a process-oriented model that simulates growth and persistence of a wide range of organisms (weeds, crops, insects, and fungi) using eco-physiologically-relevant functions based on climate variables. Because CLIMEX uses a library of well-studied population growth and stress mechanisms that draw on a solid foundation of experimental biology, parameters in CLIMEX models have biological or ecological meaning. A set of CLIMEX parameter values can be inferred from the distribution of the species by adjusting parameter values until the simulated distribution matches the known distribution in a region. The potential distribution of the organism elsewhere can then be predicted. CLIMEX has previously been used to indicate the likely severity of *Pseudocercospora* leaf disease on temperate eucalypt species [34], and to estimate the potential distribution of other pathogens [35–40].

The CLIMEX Compare Locations model [23, 24] calculates an annual index of climatic suitability, the Ecoclimatic Index (EI), which reflects the overall potential for population persistence, accounting for growth during favourable periods and survival during stressful periods (Eq 1). The annual Growth Index (GI_A_) describes the potential for growth of the modelled species as a function of average weekly soil moisture (Moisture Index; MI) and temperature (Temperature Index; TI) (Eq 2; Weekly Growth Index, GI_W_ = TI x MI). Stress indices describing cold stress (CS), wet stress (WS), heat stress (HS) and dry stress (DS), and their interactions, can be used to describe the species response to climatically unfavourable conditions. The individual components of stress are combined into a stress index (SI) (Eq 3) and a stress interaction index (SX) (Eq 4; CDX = Cold-Dry Stress, CWX = Cold-Wet Stress, HDX = Hot-Dry Stress and HWX = Hot-Wet Stress)[23].

$$EI={GI}_{A}\times SI\times SX$$ (1)

$${GI}_{A}=100\sum_{i-1}^{52}{TGI}_{W}/52$$ (2)

SI=(1−CS/100)(1−DS/100)(1−HS/100)(1−WS/100) (3)

SX=(1−CDX/100)(1−CWX/100)(1−HDX/100)(1−HWX/100) (4)

The EI ranges from 0 for locations at which the species is not able to persist, to 100 at locations that are optimal for the species year round. It is important to appreciate that the EI only has meaning as an annual summary statistic, related to population persistence. Similarly, the GI_A_ varies from 0 (no growth) to 100 (maximum/optimal growth).

In fitting parameter values, we initially concentrated on ensuring that Oceania and Southeast Asia were simulated as suitable, since this is the centre of origin of *P*. *fijiensis*, and then considered the modelled climatic suitability for location records in Central and South America. Finally, we confirmed that CLIMEX correctly simulates suitability of known locations in Africa and the Caribbean islands, before validating the model with previously unused location records worldwide. The final model parameters are given in Table 1, with an explanation of each parameter and the rationale behind each value provided below.

**Table 1:** CLIMEX parameter values for *Pseudocercospora fijiensis*.

Parameter	Description	Value
**Moisture**		
SM0	lower soil moisture threshold	0.8
SM1	lower optimum soil moisture	1
SM2	upper optimum soil moisture	2.5
SM3	upper soil moisture threshold	4
**Temperature**		
DV0	lower threshold	15°C
DV1	lower optimum temperature	25°C
DV2	upper optimum temperature	28°C
DV3	upper threshold	37°C
**Cold Stress**		
TTCS	cold stress temperature threshold	2.5
THCS	temperature threshold stress accumulation rate	-0.01 week-^1^
**Heat Stress**		
TTHS	heat stress temperature threshold	37°C
THHS	temperature threshold stress accumulation rate	0.01 week^-1^
**Dry Stress**		
SMDS	soil moisture dry stress threshold	0.5
HDS	Dry stress accumulation rate	-0.0005 week^-1^
**Threshold Heat Sum**		
PDD	number of degree-days above DV0 needed to complete one generation	1100°C-days
**Irrigation Scenario**	5 mm day^-1^, applied as top-up	

#### Soil moisture parameters

CLIMEX is a niche model, and as such, it uses the variables that in concert represent the overall climatic nature of a habitat. Soil moisture combines the interactions of temperature, rainfall and evaporation into a single variable that captures the overall moisture characteristic of that habitat. Hence, a CLIMEX model captures a species’ response to its habitat in a broad sense, rather than describing a detailed response to a single factor (i.e., leaf-wetness or dew point). Four soil moisture (SM) parameters represent how a species responds to SM: lower and upper thresholds (SM0 and SM3, respectively) define the limits within which growth can occur, and the lower and upper optimum parameters (SM1 and SM2, respectively) define the SM levels within which optimal growth rates can be achieved. This non-linear formulation accords with the Law of Tolerance [41]. CLIMEX uses a single-bucket soil moisture model, with the permanent wilting point of plants equivalent to a value of 0.1, and field capacity equivalent to a value of 1 [23]. However, pathogens often require free water for parts of their lifecycles; hence, the lower threshold (SM0) is generally set above 0.1, and the upper threshold (SM3) above field capacity to allow for run-off conditions [36–40, 42]. Moisture parameter values were derived from information available in the literature. The lower soil moisture threshold (SM0) is set to 0.8, as conidia cannot germinate below 92% RH [43] or 95% RH [44] and ascospores cannot germinate below 98% RH [43, 44]. Because optimal disease growth requires free water [44–46], the lower optimal soil moisture (SM1) is set to 1. The upper optimal value (SM2) is set to 2.5, to ensure that very wet conditions are suitable. Similarly, the upper soil moisture threshold (SM3) is set to 4, to remove any growth constraints as a result of very high rainfall.

#### Temperature parameters

As with SM, four parameters are used in CLIMEX to define a species’ response to temperature: the lower and upper thresholds (DV0 and DV3, respectively) define the limits within which growth can occur, and the lower and upper optimum temperatures (DV1 and DV2, respectively) define that range in which the maximum growth rate can be attained. Temperatures below 20°C appear to be unfavourable for the growth of various lifestages [46–48]. However, under constant temperature regimes, there is ascospore germination and growth of germ tubes at 12°C but not at 11°C [49], and other authors show ascospore and conidial germination and growth of germ tubes at temperatures below 20°C [48, 50]. These results would indicate that the lower developmental threshold could be as low as 12°C. It was also shown that under constant temperature regimes, growth occurs at half the speed at 20°C than at 27°C [49]. The optimum temperature range is between 25°C and 28°C [5, 43, 44, 46, 48, 49, 51]. Thus, whilst this information makes it reasonably straightforward to set the optimal range (DV1 = 25°C and DV2 = 28°C), it is less clear what the minimum threshold for growth should be. In CLIMEX, with a minimum threshold of 15°C and a lower optimum of 25°C, the growth rate at 20°C will be half that at the optimum, in accordance with the results of Pérez-Vicente et al. [49]. Because of this halving of the growth rate at 20°C [49], and because *P*. *fijiensis* is predominantly a tropical species and any growth below 15°C is likely to be exceedingly minimal, we opted to set the lower temperature threshold (DV0) to 15°C rather than 12°C.

Information on disease growth at high temperatures is equivocal. Whilst it has been suggested that temperatures above 35°C are unfavourable [43], a number of authors report growth of various lifestages at 35°C [44, 50, 52]. Ploetz (3) indicates that the maximum temperature for ascospore infection is 36°C, and Pérez-Vicente [49] indicates that there is some growth at 36°C but not at 37°C and 38°C. Based on this evidence, we set the maximum temperature for development (DV3) to 37°C.

#### Cold stress

Cold stress is used to restrict the pole-ward extent of the distribution. As a wet-tropical species, it is unlikely that *P*. *fijiensis* is frost-tolerant. A temperature threshold of 2.5°C in a climate dataset might allow for several days in a week where temperatures drop to 0°C [53], and a frost-sensitive species may be limited to areas where the long-term average minimum temperature is above 2.5°C, rather than 0°C [54]. For these reasons, we set the cold stress temperature threshold (TTCS) to 2.5°C, and set the stress accumulation rate (THCS) to a relatively high value of -0.01 week^-1^.

#### Heat stress

Despite the conflicting information regarding the upper temperature threshold for development (see above), it is nonetheless clear that high temperatures are detrimental to the continued growth of *P*. *fijiensis*, and so we used a temperature threshold mechanism to impose heat stress. Because heat stress cannot begin to accumulate at temperatures that are favourable to growth [23, 29], we use the upper temperature threshold (DV3) of 37°C as the threshold (TTHS) from which stress can begin to accumulate, and we set the accumulation rate (THHS) to 0.01 week^-1^.

#### Dry stress

Dry stress was included as *P*. *fijiensis* requires extended periods of leaf wetness for growth. As stated previously, CLIMEX uses soil moisture to integrate the effects of temperature, rainfall and evaporation, so leaf wetness *per se* cannot be modelled. However, to simulate an extended period of dryness, we used a low rate of stress accumulation. With a threshold soil moisture (SMDS) of 0.5 and a stress accumulation rate (HDS) of -0.0005 week^-1^, a very small amount of dry stress occurs in Southeast Asia and the tropics of Africa, but the level is insufficient to have any impact on the growth of *P*. *fijiensis* in these areas.

#### PDD, threshold heat sum, or degree-days per generation

In addition to stresses accumulating to such high levels that population persistence at a location may be precluded (see Eq 2 above), population persistence may also be precluded if a species does not accumulate enough thermal energy during the growth season to complete a minimum amount of development (usually one generation). This is generally apparent at high altitudes and latitudes. In CLIMEX, the threshold heat sum is used to define the number of degree-days (PDD) of thermal accumulation above DV0 (the development threshold) required by a species to complete a generation. If this threshold value is not reached, there is insufficient warmth to allow the lifecycle to be completed in a year, and species establishment is prevented. As we were not able to locate good information with which to be able to set this parameter, we used location records to fit an appropriate value. Although it has been shown that *P*. *fijiensis* can persist in the absence of sexual reproduction, and hence without a complete sexual lifecycle [55, 56], these studies both assume optimal growth conditions and therefore cannot be used to help set a value for PDD.

In Brazil, *P*. *fijiensis* occurs in the municipalities of Cristina (altitude between 950 m and 1 500 m), São Jose do Alegre (altitude between 800 m and 900 m), Gonçalves (altitude between 1 200 m and 1 400 m) and Piranguçu (altitude between 860 m and 940 m) [2]. As we geo-coded these locations at town centres, and we do not know exactly where the sampling was conducted, we need to ensure that the area around these location records is suitable. In the CliMond data set, Cristina falls near the north edge of a grid cell with an altitude of 1 339 m, which correctly represents the area, and accumulates 1 129 degree-days above 15°C (DV0). The town of Gonçalves falls in the southeast corner of a grid cell, with an altitude of 1 111 m, accumulating 1 251 degree-days. With a PDD value of 1 100 degree-days, *P*. *fijiensis* can persist in the immediate vicinities of both Cristina and Gonçalves. A higher PDD of 1 200 would preclude persistence in the grid cell within which Cristina falls, but would allow persistence in the lower-altitude regions surrounding the town. Although Google Earth shows that the area surrounding both towns is intensively cultivated, it is not possible to identify where banana plants are grown. Thus, we opt for a more conservative approach and use a value of 1 100 degree-days, even though we could be marginally overestimating the potential range of *P*. *fijiensis*.

#### Irrigation scenario

Banana production demands high soil moisture levels for optimum growth (S1 Table). A summary of the literature suggests that banana crops use as much water as is lost by evapotranspiration [57], thereby indicating that crop water requirements depend on the location and the season, varying between 3–14 mm day^-1^ (S1 Table). To assess the impact of irrigation in regions where it is practiced [33], we used an irrigation scenario of 5 mm day^-1^ (equating to an annual rainfall of 1 825 mm) applied as top-up, so that irrigation is only applied when natural rainfall does not reach this amount. This amount of irrigation is at the low end of the recommendation for growing banana plants (S1 Table), but it removes virtually all dryness constraints in irrigated areas, leaving only temperature to regulate the growth of *P*. *fijiensis*.

### Parameter sensitivity and model uncertainty

CLIMEX Version 4 provides the option to undertake automated sensitivity and uncertainty analyses [23]. The sensitivity analysis identifies the degree to which each parameter affects the projected total area of suitable habitat and other model state variables. The result of the one-parameter-at-a-time sensitivity analysis is a table of values indicating relative sensitivity of each parameter for each state variable. The uncertainty analysis accounts for the fact that the parametric uncertainty affects all of the parameters simultaneously. It uses a Latin hypercube to sample a triangular distribution of values spanning each of the default parameters used in the species parameter file. To address the anisotropism between variables, the uncertainty bounds are scaled differently for each parameter type. Thus, the results reflect a general sense of uncertainty associated with our ability to estimate each of the parameter types, rather than any specific consideration of our confidence in our ability to estimate each specific parameter [23]. The result of the uncertainty analysis is an agreement map indicating the proportion of models from the Latin hypercube sampling (n = 50) that resulted in a suitable Ecoclimatic Index value (EI > 0). For each of the sensitivity and uncertainty analyses, the default model parameters were run using the same CM10 1975H V1.1 dataset used for the model fitting process. The analyses were only performed for the natural rainfall scenario. The results of both the sensitivity and uncertainty analyses depend on the region over which the analyses are run. In this case, the analyses were run for the entire world.

### Qualitative ranking of disease pressure

Expert opinion was elicited in a global black leaf streak workshop held at Guápiles, Costa Rica, in 2008. Ten BLSD expert researchers were asked, individually, to pinpoint locations in Google Earth where they had conducted field work and/or had performed field visits, and they knew *P*. *fijiensis* was present. There were no constraints placed on the number of visits or how recent the visits were to filter out experts or sites per expert, but it was clear that experts had been visiting these locations at least once per year during the previous three to five years. Some experts only rated the country or locality where they were based at the time of the workshop. Once experts had selected the locations of *P*. *fijiensis* presence, they were asked to rate the sites according to a disease pressure (DP) score, which ranged from 1 (lowest pressure) to 10 (highest pressure). Since all locations were commercial farms cultivated mostly with Cavendish cultivars which are all susceptible to BLSD, the DP score is unlikely to be biased by varietal resistance to *P*. *fijiensis*. This dataset comprised of 88 localities from 20 countries. All six experts were advised orally at the workshop that their participation in the workshop and provision of their assessments comprised consent to use this information in the development of a publishable manuscript.

### Potential disease pressure and production

The relationship between DP and EI allows us to examine the likely risk exposure patterns for banana and plantain production. The expert ranking of disease pressure was statistically modelled in relation to the CLIMEX EI value. The nlstools package (1.0–2) in R (version 3.3.1) was used to fit a rectangular hyperbolic function (Eq 5) to the data:

$$\bar{I_{DP}}=\frac{A∙EI}{K+EI}$$ (5)

Where

$\bar{I_{DP}}$ is the average disease pressure index estimated by expert elicitation,

*A* is the asymptote, and

*K* is the EI value when $\bar{I_{DP}}$ equals *A*/2.

To assess the distribution of *P*. *fijiensis* modelled risk to banana production we used ArcMAP (ESRI) to spatially-intersect the CLIMEX EI results with banana production data from MapSpam [58]. The MapSpam data were firstly spatially aggregated from 5’ to 10’ to conform with the available CLiMond climatic data [28]. The fitted DP function (Eq 6) was applied to calculate an estimated DP index score at each cell. We then used the R package to calculate and plot cumulative banana production as a function of the calculated disease pressure index under a natural rainfall scenario. For each DP value, we calculated the total banana production (tonnes per year) that experience that disease pressure or less.

To explore the relationship between disease pressure and productivity, we plotted calculated DP against yield for banana and plantain. We used contour plots to expose the patterns in these bivariate data.

## Results

### Global distribution

#### Oceania and Asia

**Fig 2:**
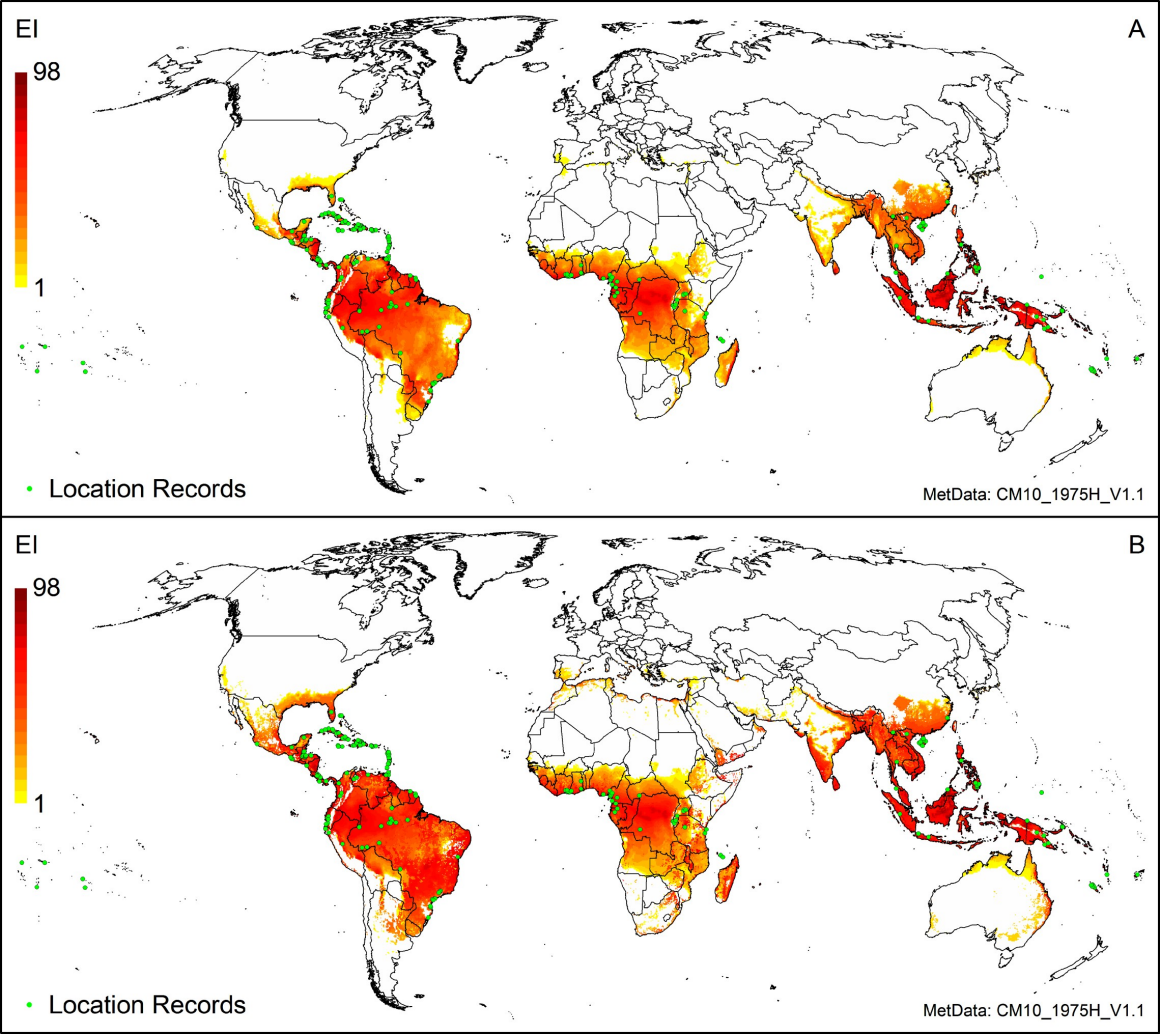
Modelled global climate suitability for *Pseudocercospora fijiensis* (A) under a natural rainfall scenario, and (B), mapped as a composite of natural rainfall and irrigation based on identified irrigation areas [33].

The CLIMEX model projects all of the islands of Oceania to be highly suitable (Fig 2, S2 Fig). As expected, tropical islands (e.g., Philippines, Indonesia, Papua New Guinea, Timor-Leste) are projected to be more suitable than sub-tropical continental (e.g., China, Vietnam, Thailand, Bhutan, Myanmar) and high altitude (e.g., Nepal, northern India) areas. Banana production in sub-tropical Taiwan is seasonal, with most bananas harvested between March and June [59, 60]. In southern Taiwan, *P*. *fijiensis* first appears in early May and continues throughout the summer [61], suggesting that the banana plants are available beyond June, and CLIMEX growth charts for this area support these findings (S3 Fig). Similar seasonality is observed in Fiji, with CLIMEX weekly growth index charts showing reduced growth of *P*. *fijiensis* in the cooler months of July and August, which correlates with Wardlaw [62] (S4 Fig).

The Philippines, which has a tropical temperature pattern, appears entirely suitable under a natural rainfall scenario (S2 Fig). This accords with published information, which also indicates that black Sigatoka disease is found in all Cavendish plantations, and is present on leaves all year round [12]. Only two high-altitude (>1 740 m) grid cells in the southwest corner of the Mountain Province on the main island are unsuitable, having insufficient growing degree-days with which to complete a generation. Crop distribution data [58] indicates that banana in the Philippines is grown both under natural rainfall conditions and under irrigation. Areas experiencing drier periods become more suitable for *P*. *fijiensis* with the irrigation scenario; nonetheless, natural rainfall conditions are sufficient to enable this species to persist throughout the Philippines.

As stated above, *P*. *fijiensis* grows in other countries in south-east Asia (China, Indonesia, Malaysia, Singapore, Thailand and Vietnam) [5, 21, 22, 63]. The CLIMEX model projects these areas as suitable under a natural rainfall scenario, and even more suitable under the top-up irrigation scenario (Fig 2). *P*. *fijiensis* is recorded from southwest Bhutan, but with no additional details as to the location [64], and the model projects the southern part of Bhutan to be suitable, at altitudes ≤ 1 665 m.

In south Asia, there are suggestions in the literature of *P*. *fijiensis* occurring in India [64–66]. However, molecular techniques indicate that the species present in both India and Sri Lanka is *P*. *eumusae*, not *P*. *fijiensis* [67–69]. Much of the central region of India is projected as unsuitable, largely due to excessive heat stress. Banana in India is grown under natural rainfall conditions [58, 70], and all production areas are projected to be climatically suitable for *P*. *fijiensis* (Fig 2A). Apart from a single unsuitably cold and high-altitude grid cell (1 723 m), all of Sri Lanka is projected to be suitable.

#### Australia

Across Australia, suitability is projected to be much lower than in Asia (Fig 2, S2 Fig). *Pseudocercospora fijiensis* is prevalent in the Torres Strait Islands, and all incursions on mainland Australia (in northeast Queensland) have resulted in successful eradication efforts [15, 71, 72]. Banana production in Australia generally requires irrigation [12, 73].

A region of suitable climate extends down along the eastern seaboard, to just south of Sydney (Fig 2; S5 Fig). This area encompasses all regions in eastern Australia where banana plants are grown commercially. Kununurra, in Western Australia, where some banana is grown [12], experiences excessive heat stress (HS = 126), and *P*. *fijiensis* is not able to persist (EI = 0), although growth can occur in the cooler months of February to September if irrigation is increased to 7 mm day^-1^ as top-up (S6 Fig). Although Carnarvon in Western Australia does not appear to be suitable under any irrigation scenario in these maps (Fig 2; S5 Fig), this would appear to be an issue with the irrigated areas data [33], as growth is projected to occur with irrigation (S7 Fig), and banana is indeed grown here under irrigation [12].

#### Latin America and the Caribbean

All of Central America is projected to be climatically suitable for the persistence of *P*. *fijiensis* under a natural rainfall scenario (Fig 2, S8 Fig), apart from some high-altitude regions (> 1 500 m) in Costa Rica, Guatemala and Mexico. Where the irrigation scenario is applied, these areas become more suitable and growth of *P*. *fijiensis* becomes temperature-limited (e.g., Guatemala, Honduras, Nicaragua, S9–S11 Figs). Costa Rica is the second exporter of commercial bananas in Latin America [15], with major producing areas along the Atlantic coast. In these areas, BLSD disease pressure is high, and fungicides are applied up to 45 times per year [1]. CLIMEX predicts climatic suitability for *P*. *fijiensis* to be very high (EI > 70).

In South America, the major export banana cultivation areas of northern Colombia, Ecuador and Peru appear to be less climatically suitable for *P*. *fijiensis* than Central America (Fig 2, S8 Fig). All location records in South America fall within suitable climate space under a natural rainfall scenario, apart from three sites in north-eastern Peru (in the departments of Tumbes and Piura) which are too dry for *P*. *fijiensis* (EI = 0, Fig 2A, S8 Fig, S12A Fig). Agriculture here relies upon irrigation [74, 75]. Whilst the irrigation scenario of 5 mm day^-1^ as top-up makes all these locations highly suitable (EI ≥ 60, Fig 2B, S12B Fig), the incidence of BLSD is low (see S12 Fig). Most of Brazil is projected to be suitable for *P*. *fijiensis*, with irrigation enhancing suitability (S12 Fig).

Across the Caribbean, there are also major hotspots of *P*. *fijiensis* suitability. Most of Haiti is suitable for *P*. *fijiensis* under a natural rainfall scenario, and all location records fall within grid cells where EI > 40 (Fig 2A, S13A Fig). The model is consistent with the observation that the disease is more prevalent, with higher levels of damage, in the wetter areas of Haiti (CA, pers. obs.). Irrigation is widely used for banana cultivation in Cuba [76], and for banana and plantain production in the Dominican Republic and Jamaica [77] and in Puerto Rico [78]. While these four countries are suitable for *P*. *fijiensis* under a natural rainfall scenario (Fig 2A, S13A Fig), they become even more suitable with irrigation (Fig 2B, S13B Fig), as do Antigua and Barbuda, Guadeloupe, Grenada, and Trinidad and Tobago. However, irrigation does not improve the suitability of other Caribbean islands for which there are location records: Dominica, Martinique, St. Lucia, and St. Vincent and the Grenadines.

#### Africa

The modelled climate suitability of Africa under a natural rainfall scenario captures all of the geocoded location records (Fig 2A, S14A Fig), as well as all countries listed as having *P*. *fijiensis* [5], and suitability is enhanced with irrigation in those regions where it is practiced [33] (Fig 2B, S14B Fig).

#### USA

*Pseudocercospora fijiensis* has been detected in both Hawaii and Florida [5], and both these areas are climatically suitable (Fig 2). In these regions, banana plants are not cultivated in plots, but rather in backyards near houses.

#### Validation

All 119 location records used to validate the model fall within cells modelled as being climatically suitable. Most location records fall in cells that are climatically suitable under natural rainfall conditions; however, the few validation records collected from irrigated sites all fall within a suitable climate space under the irrigation scenario (Fig 2). The validation records thus all indicate that our model is robust.

### Host masking

**Fig 3:**
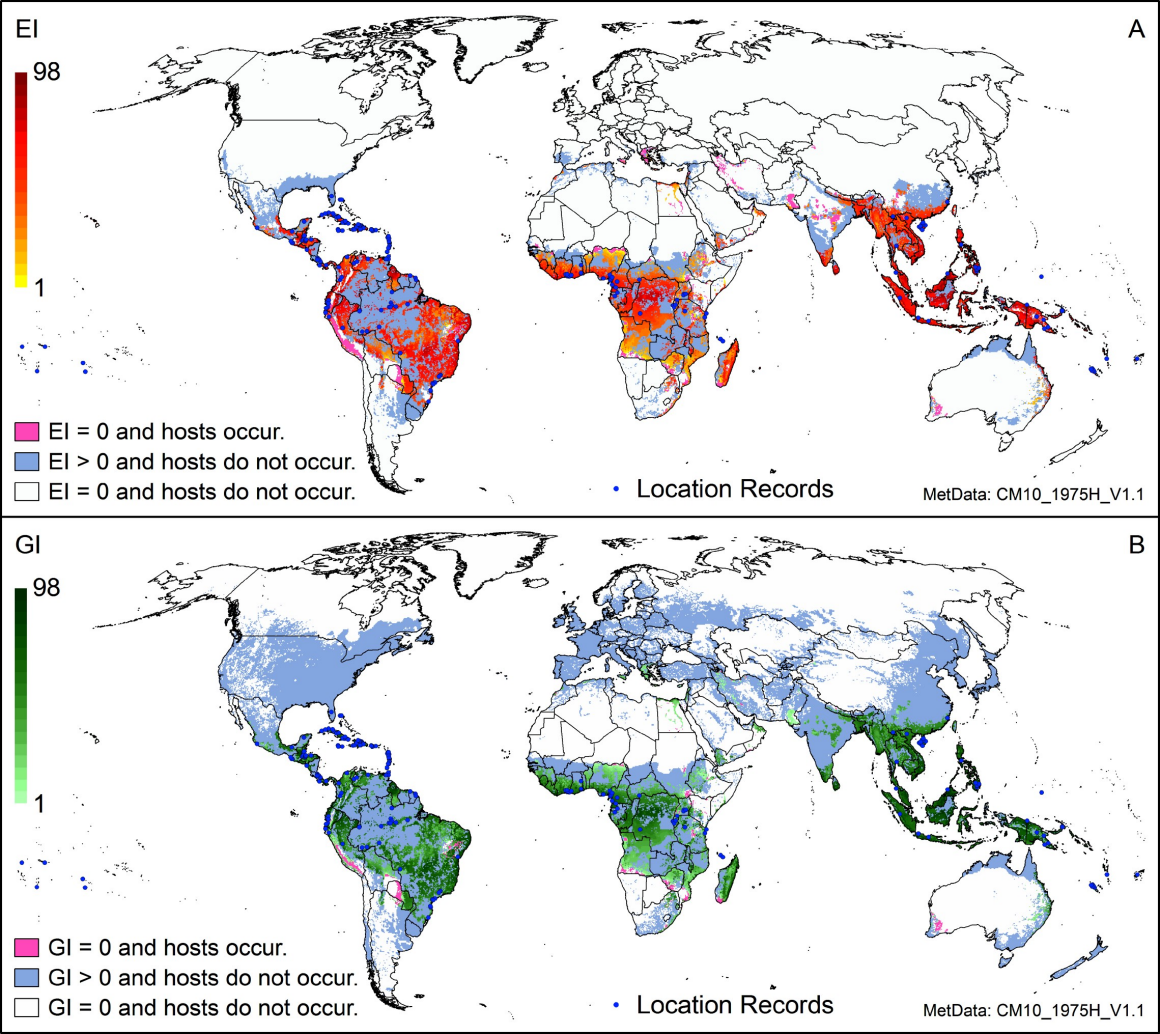
Modelled global climate suitability for *Pseudocercospora fijiensis* (A) to persist (EI), mapped as a composite of natural rainfall and irrigation based on identified irrigation areas [33], then masked by harvested areas of host plants (banana, plantain) [58] and (B) to have positive growth (GI) in harvested areas of host plants under an irrigation scenario regardless of the potential to persist as a permanent population.

We masked the CLIMEX composite output by host availability, using available production area data [58] (Fig 3). This figure very clearly identifies areas where banana and/or plantain are currently grown, but which are climatically unsuitable for the persistence (EI) of growth (GI) of *P*. *fijiensis* (pink areas). Most notably, the areas unsuitable for persistence include the Peruvian coast, the northern part of Nigeria, parts of East Africa, Portugal, Sicily, and isolated areas of the Middle East (Israel, Yemen, Oman, Iran), Asia (Pakistan, India and China), and Western Australia (Fig 3A). However, many of these areas are modelled as suitable for temporary populations of *P*.*°fijiensis*, with population growth possible at suitable times of the year (Fig 3B), thus highlighting areas at potential risk. Fig 3 also highlights those regions into which *P*. *fijiensis* could spread, were banana and/or plantain production areas to be expanded (grey areas). A prime example is the validation record for Florida (USA): *P*. *fijiensis* has been collected here, but because banana plants are only cultivated in backyards, there is no official production data.

### Parameter sensitivity and model uncertainty

Parameter sensitivities are presented in Table 2, with the model parameters listed in descending sensitivity for modelled range. Even the most sensitive parameter (heat stress temperature threshold, TTHS) only has less than a 1% impact on the potential range.

**Table 2:** CLIMEX parameter sensitivity values for *Pseudocercospora fijiensis* parameters listed in Table 1, as applied to the CM10 1975 V1.1 global dataset under a natural rainfall scenario.

		Parameter Values			% Range	EI	% Core Distribution	Growth Variable Changes			Stress Variable Changes		
Parameter	Mnemonic	Low	Default	High	Change	Change	Change	MI	TI	GI	CS	HS	DS
Heat stress temperature threshold	TTHS	36	37	38	0.71	1.54	0.00	0	0	0	0	19.24	0
Limiting low temperature	DV0	14	15	16	0.46	1.95	0	0	2.17	1.00	0	0	0
Degree-days per generation	PDD	880	1100	1320	0.42	1.61	0	0	0	0	0	0	0
Limiting low moisture	SM0	0.7	0.8	0.9	0.30	0.91	0	5.33	0	1.02	0	0	0
Cold stress temperature threshold	TTCS	1.5	2.5	3.5	0.26	1.59	0.39	0	0	0	14.60	0	0
Heat stress temperature rate	THHS	0.008	0.01	0.012	0.16	0.31	0	0	0	0	0	3.76	0
Lower optimal moisture	SM1	0.9	1	1.1	0.08	0.90	0	5.34	0	1.00	0	0	0
Cold stress temperature rate	THCS	-0.012	-0.01	-0.008	0.06	0.34	0.01	0	0	0	2.88	0	0
Lower optimal temperature	DV1	24	25	26	0.02	2.14	0	0	4.27	2.24	0	0	0
Limiting high temperature	DV3	36	37	38	0.01	1.54	0	0	3.96	1.59	0	0	0
Upper optimal temperature	DV2	27	28	29	0.01	3.68	0	0	5.60	3.75	0	0	0
Dry stress threshold	SMDS	0.4	0.5	0.6	0.01	0.35	1.42	0	0	0	0	0	31.50
Dry stress rate	HDS	-0.0006	-0.0005	-0.0004	0.01	0.24	0.47	0	0	0	0	0	11.73
Upper optimal moisture	SM2	2.4	2.5	2.6	0.00	0.07	0	0.09	0	0.08	0	0	0
Limiting high moisture	SM3	3.9	4	4.1	0.00	0.04	0	0.04	0	0.04	0	0	0

**Fig 4:**
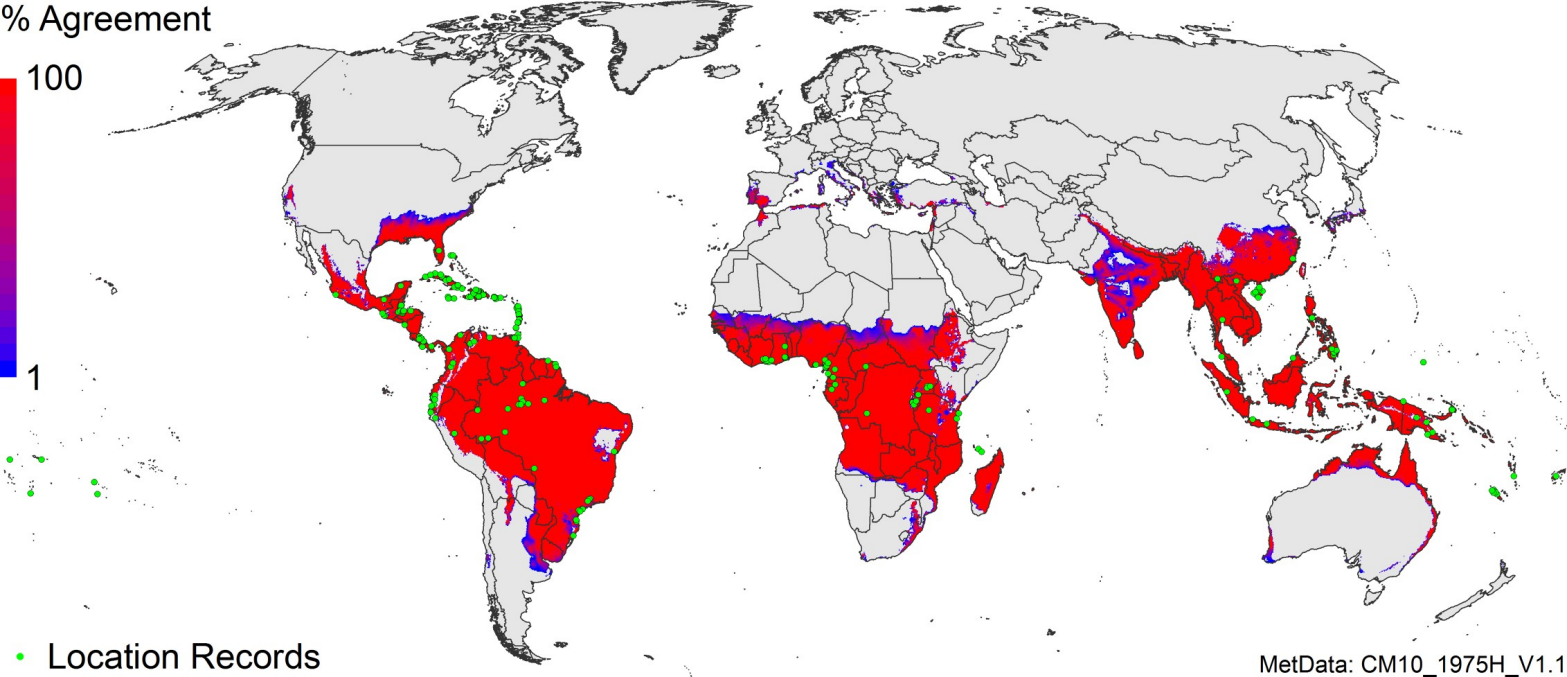
CLIMEX parametric uncertainty analysis for *Pseudocercospora fijiensis* run under the natural rainfall scenario. The fitted parameters were systematically modified to reflect uncertainty. The plotted values represent the percentage of model runs with an EI value greater than or equal to one.

The parametric uncertainty of the model is portrayed in Fig 4. Blue areas indicate that only very few (extreme) parameter combinations indicate the potential for establishment, and are therefore unlikely. The limited extent of these blue areas indicates that the fitted parameter set is highly stable. It would take a gross error in the fitted parameters (or the climatological dataset) for these marginal areas to be suitable under current climate and irrigation patterns.

### Comparison of climate suitability with expert ratings of disease pressure

**Fig 5:**
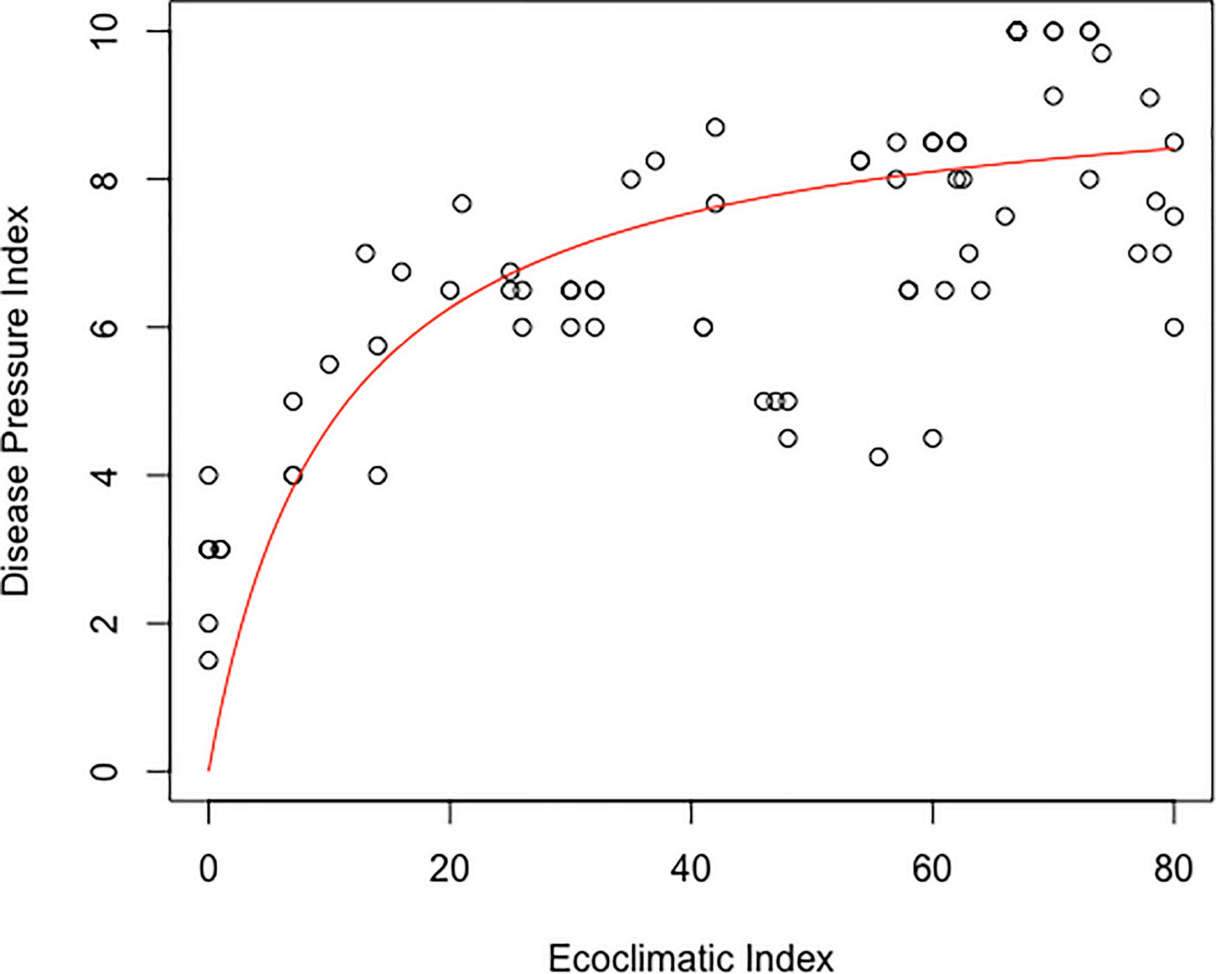
Fitted value of average disease pressure index evaluated by expert opinion as a function of the modelled CLIMEX Ecoclimatic Index (EI) under a natural rainfall scenario. R^2^ = 0.577 (n = 88).

There is a moderately strong non-linear relationship between expert scoring of disease pressure (DP) with CLIMEX estimates of EI (Fig 5). The relationship increases strongly at low values of EI, and steadies at higher values. At the higher EI values, variance in DP values increases. The EI value explains 58% (p<0.001) of the variation in expert-based disease pressure rankings: the rectangular hyperbolic regression function fitted to the data (Fig 5) has an R^2^ value of 0.577 (n = 88), as per Eq 6:

$$\bar{I_{DP}}=\frac{9.5∙EI}{10.36+EI}$$ (6)

### Potential disease pressure and production

**Fig 6:**
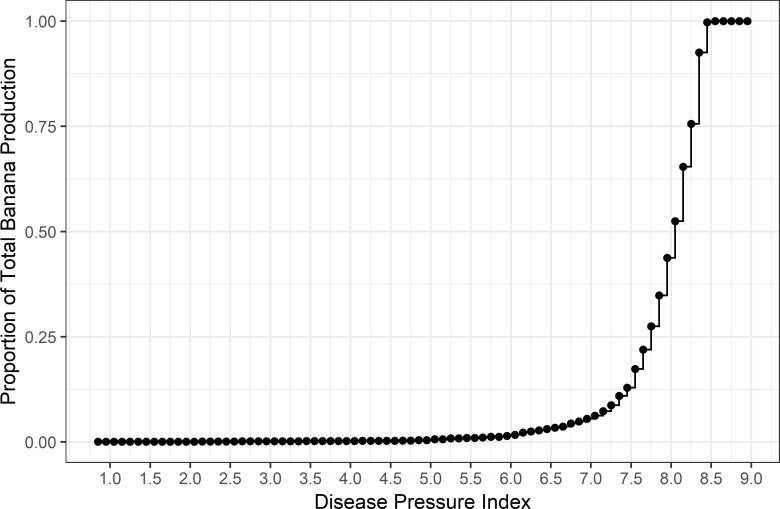
Fitted value of cumulative banana production as a function of the calculated disease pressure index under a natural rainfall scenario.

**Fig 7:**
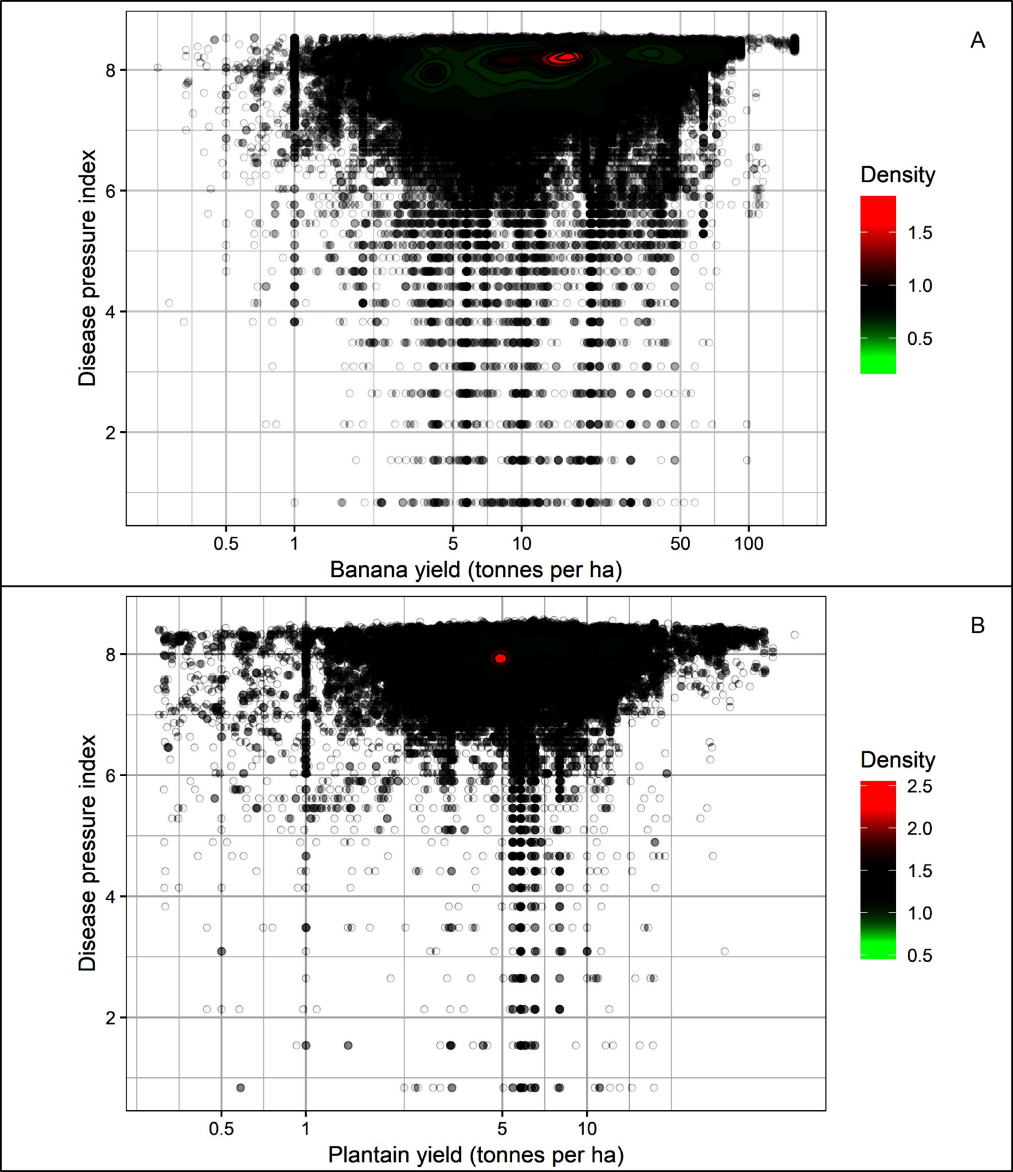
Contour plot showing the relationship between calculated disease pressure index and yield of (A) banana and (B) plantain under a natural rainfall scenario.

Most banana production occurs in areas where the estimated DP is highest (Fig 6). The greatest proportion of global banana production (94.5%) occurs in areas which experience an estimated DP value of 7 or above. Furthermore, the higher yielding production areas for both banana and plantain experience the highest potential disease pressure (Fig 7).

## Discussion

CLIMEX was used to build a niche model for *P*. *fijiensis*, to assess the potential distribution and the areas at risk of this important pathogen. This is the first occasion where known information on the ecology, biology and global distribution of *P*. *fijiensis* has been collated. It also is the first attempt to create a potential distribution map, and more importantly, to understand what drives the spatial distribution of BLSD. Recent studies on the spread of BLSD in the absence of sexual spore production [55, 56] do not assist such a mapping exercise, and despite decades of research and the increasing importance of BLSD to the banana industry globally, the spatial distribution of *P*. *fijiensis* is not known with any certainty. The CLIMEX model was also used to gain an insight into the level of disease pressure exerted on banana and plantain production and yield.

Published data were used to set most of the soil moisture and temperature parameters in our model, and we used the literature to guide the choice of those values. In other instances, we used distribution data to fit parameter values [e.g., PDD was set to enable persistence in known locations (Brazil)]. It is this ability to define parameter values from a variety of information sources that sets CLIMEX apart from correlative species distribution models (SDMs): CLIMEX combines the strengths of correlative modelling methods (allowing stress parameters to be fitted to geographical location data) and deductive modelling methods (allowing growth functions to be informed by laboratory studies and phenological observations) [23]. The modelled potential distribution encompasses all known locations where *P*. *fijiensis* has been collected, including all of the validation sites that were not used to construct the model. This suggests that the model parameters are reasonable and correctly reflect the interaction of the pathogen with its environment. CLIMEX predictions also compare well with expert scores of BLS disease pressure.

Parameter sensitivity analysis does not highlight any concerns with parameter values, as the most sensitive parameters have less than a 1% impact on the modelled potential range (Table 2). The most sensitive parameter (heat stress temperature threshold, TTHS), only has a 0.71% impact on this species’ potential range when tested over a 2°C range of values. We have a high degree of confidence in the upper temperature threshold value (DV3 = 37°C, see Methods, setting temperature parameters). In CLIMEX, temperature stresses cannot accumulate within the range of temperatures suitable for growth [23, 29, 30, 54, 79]. Therefore, heat stress can only accumulate at or above the upper developmental threshold, and so the high degree of confidence in our upper temperature threshold for growth (DV3) also provides us with a high degree of confidence in the TTHS value. The lower temperature threshold (DV0) is the next most sensitive parameter (0.46%). Whilst DV0 may be as low as 12°C [49], the literature suggests that temperatures below 20°C are unfavourable for growth [46–48], and so again, we can be reasonably confident that our DV0 value is suitable. The third most sensitive parameter (0.42%) is the number of degree-days per generation, PDD. The value of this parameter is necessarily linked to DV0, so it is not altogether surprising that these two parameters have a similar impact on the range. We set the PDD value to enable *P*. *fijiensis* to persist in known locations in Brazil [2], and the relatively wide range over which this value was tested (880–1320 degree-days) only has a small impact (0.42%), giving us confidence that our value need only be approximately correct for the model results to remain stable. All other parameter values have a 0.3% or less sensitivity to the modelled range, which is negligible. Any changes in the Growth Index (GI) reflect changes in the Moisture or Temperature Indices (MI or TI), and changes in the stress indices reflect a sensitivity to their corresponding parameters, all of which merely highlight the internal consistency of the CLIMEX model.

The uncertainty map corroborates the robustness of the model under a natural rainfall scenario, by highlighting those geographical areas where we have greater or lesser confidence in the model performance (Fig 4). Model robustness was further substantiated by explicitly examining the role of irrigation in modifying the suitability of locations, as previously demonstrated [29, 31, 32, 79]. The moderate irrigation scenario that we use (5 mm day^-1^ as top-up) removes all moisture deficits, making growth of *P*. *fijiensis* temperature-dependent. In some of these areas (e.g., the Caribbean), the modelled growth of *P*. *fijiensis* remains sub-optimal due to the temperature conditions (S13 Fig), and is manifested by a low disease pressure in Gonaives (Haiti) and Valverde (Dominican Republic), and the observation that yellow Sigatoka is more predominant than BLSD in Azua (Dominican Republic) (CA, pers. obs.).

We noticed that some sites had a low DP rating despite having a high EI under irrigated conditions. In an attempt to understand this situation we analysed the temperature (S15 Fig) and soil moisture profiles (S16 Fig) for the ten locations (in Belize, Costa Rica and Honduras) with the highest disease pressure (DP = 10) and the five locations (in the Dominican Republic and Peru) with the lowest disease pressure (DP ≤ 3). Apart from two relatively cool locations in Peru, there is nothing in the temperature patterns to differentiate these two sets of sites (S15 Fig). Soil moisture, on the other hand, is much lower in the low-ranked sites than in the high-ranked sites (S16A Fig). The irrigation scenario is sufficient to remove all moisture limitations, making the soil moisture profiles of all sites comparable (S16B Fig) and increasing the EI values of the five lowest-ranked sites to levels comparable to the highest-ranked sites (see EI values in brackets in S15 and S16 Figs). Whilst the irrigation scenario removes all moisture deficits, it may not correctly reflect atmospheric moisture or humidity, which will determine whether or not banana leaves get wet enough for long enough to support development. These results highlight a strategic management opportunity, which is likely already being implemented in Peru [74, 75] (S12 Fig) and Dominican Republic [77] (S13 Fig). Disease pressure in these regions is acknowledged to be low, and it appears that irrigation via flooding or drip system provides the water necessary for banana plants to grow without increasing the atmospheric humidity to levels that become very suitable for *P*. *fijiensis*. Whilst it is beyond the scope of this paper, it would be possible to use this information in CLIMEX to identify novel regions for banana production under a specified irrigation scenario.

The CLIMEX EI values appear to be related in a non-linear fashion to expert DP ratings. Differences in DP rankings may be due to atmospheric humidity (as discussed above) or to host suitability effects. Nonetheless, the perceived severity of disease is well-characterised by the CLIMEX model (Fig 5). The non-linear fitted relationship means that at low EI values the DP index increases strongly with EI, and this rate of increase declines steadily as EI increases further. This could suggest that the disease pressure is steadily saturating as climatic conditions become ever more suitable, or that the DP scale itself is non-linear. Irrespective of the cause, it is difficult to discriminate higher values of DP score based upon modelled climate suitability above EI values of approximately 30.

We used the fitted relationship between EI and DP (Eq 6) to examine the relationship of estimated DP with production and yield of banana and plantain. All our analyses (Figs 6 and 7) indicate a strong relationship between production and DP: areas of highest production (tonnes) have the highest DP (Fig 5), and the highest density of points are in areas of both high yield (tonnes hectare^-1^) and high DP (Fig 6).

The CLIMEX Compare Locations model only addresses the interaction of *P*. *fijiensis* with climatic variables: it does not consider any species interactions (e.g., possible competition with yellow Sigatoka), or other abiotic factors. Nonetheless, it appears to correctly model the situations observed in the field. For example, where *P*. *fijiensis* is noted to be less prevalent than yellow Sigatoka (Azua, Dominican Republic, CA pers. obs.), CLIMEX indicates that the growth conditions are suboptimal for *P*. *fijiensis*. In Cameroon, *P*. *fijiensis* is restricted to areas below 1 300 m altitude, and it has been suggested that it is outcompeted by *P*. *musicola* under these cooler high-altitude conditions [80]. The current CLIMEX model presently ignores such biotic interactions, and projects the highlands of Cameroon to be climatically suitable, since attempting to model the highlands of Cameroon as unsuitable conflicts with knowledge of the distribution of *P*. *fijiensis* in tropical highland regions of Brazil [2]. With more data for locations where both pathogens co-exist, it would be possible to use the 2-species interaction model in CLIMEX to examine the relative suitability of locations for each of the species, and to explicitly examine competition between them [23].

CLIMEX is a semi-mechanistic model describing the interaction of a species with its environment, rather than merely describing the climate in which the species occurs as do correlative species distribution models [26, 81]; hence, it is a suitable platform with which to examine the potential distribution of an organism in novel climates–i.e., under climate change, as has been done for other species [37, 82, 83]. Although it is beyond the scope of the current paper, it would be possible to project disease presence and severity in countries still free of *P*. *fijiensis*, and to estimate an approximate date of occurrence in these countries based on information in [55].

Bioclimatic niche models have become popular tools used in formal pest risk assessments (PRA) within the international phytosanitary framework that underpins global trade [84, 85]. The models tend to do a good job of defining the potential geographical range of the pests, as determined from a comparison of the models with distribution data. Within the potential range, the link between modelled climate suitability and pest impacts is generally implicitly assumed to be positive, though there are few examples where the relationship has been tested [86–89]. The comparison between the expert-elicited DP scores and the modelled climate suitability patterns adds to our understanding of the relationship between modelled climate suitability and pest impacts. The goodness of fit of the regression (Eq 1) implies that the CLIMEX model is robust, at least within the bounds afforded by the expert elicitation process.

This analysis has resulted in a better understanding of the underlying factors that determine the spatial distribution of *P*. *fijiensis* globally, has enabled us to map for the first time the potential geographical distribution of this important plant pathogen, and has also enabled us to examine the relationship between agricultural production and perceived disease pressure.

## Ethics statement

The survey component of this study was carried out in 2008, at a global black leaf streak workshop held at Guápiles, Costa Rica. All participants were advised orally at the workshop that their participation in the workshop and provision of their assessments comprised consent to use this information in the development of a publishable manuscript.

## Supporting information

S1 Fig Location records for *Pseudocercospora fijiensis* used in the analysis. (PDF)

S2 Fig Modelled climate suitability under a natural rainfall scenario of Oceania and Southeast Asia for *Pseudocercospora fijiensis*. (PDF)

S3 Fig Growth charts of *Pseudocercospora fijiensis* for two grid cells in the area given by Chuang and Jeger (61) in southern Taiwan. (PDF)

S4 Fig Growth charts for two locations in Fiji. (PDF)

S5 Fig Composite map of the modelled climate suitability of Australia for *Pseudocercospora fijiensis*, based on identified irrigation areas [33]. (PDF)

S6 Fig Growth chart for *Pseudocercospora fijiensis* in Kununurra, Western Australia. (PDF)

S7 Fig Growth charts for *Pseudocercospora fijiensis* in Carnarvon, Western Australia. (PDF)

S8 Fig Modelled climate suitability under a natural rainfall scenario of Central America and the Caribbean for *Pseudocercospora fijiensis*. (PDF)

S9 Fig Growth chart for *Pseudocercospora fijiensis* in an area representative of Omagua Farm, Guatemala, and expert database site #72. (PDF)

S10 Fig Growth charts for *Pseudocercospora fijiensis* in two areas representative of Omonita Farm, Honduras. (PDF)

S11 Fig Growth chart for *Pseudocercospora fijiensis* at expert database site #80 in Nicaragua. (PDF)

S12 Fig Modelled climate suitability of South America for *Pseudocercospora fijiensis*. (PDF)

S13 Fig Modelled climate suitability of the Caribbean for *Pseudocercospora fijiensis*. (PDF)

S14 Fig Modelled climate suitability of Africa for *Pseudocercospora fijiensis*. (PDF)

S15 Fig Minimum and maximum temperatures of the CLIMEX grid cells containing the ten highest-ranked locations (lines with markers) and the five lowest-ranked locations (solid lines, no markers) of *Pseudocercospora fijiensis*. (PDF)

S16 Fig Soil moisture values of the CLIMEX grid cells containing the ten highest-ranked locations (lines with markers) and the five lowest-ranked locations (solid lines, no markers) of *Pseudocercospora fijiensis*. (PDF)

S1 Table Water requirements for optimal banana production. (PDF)

S1 File Expert scores used in the analyses. (XLSX)
