# Supplementary material for: Black Sigatoka in bananas: Ecoclimatic suitability and disease pressure assessments
Source: PLoS One. 2019 Aug 14;14(8):e0220601. doi: 10.1371/journal.pone.0220601 (PMC6693783; doi:10.1371/journal.pone.0220601)

Figure S1. Map showing the source of the location records used in the analysis. Red are the expert database records, blue are those geo-coded from the literature, and green are the validation records.

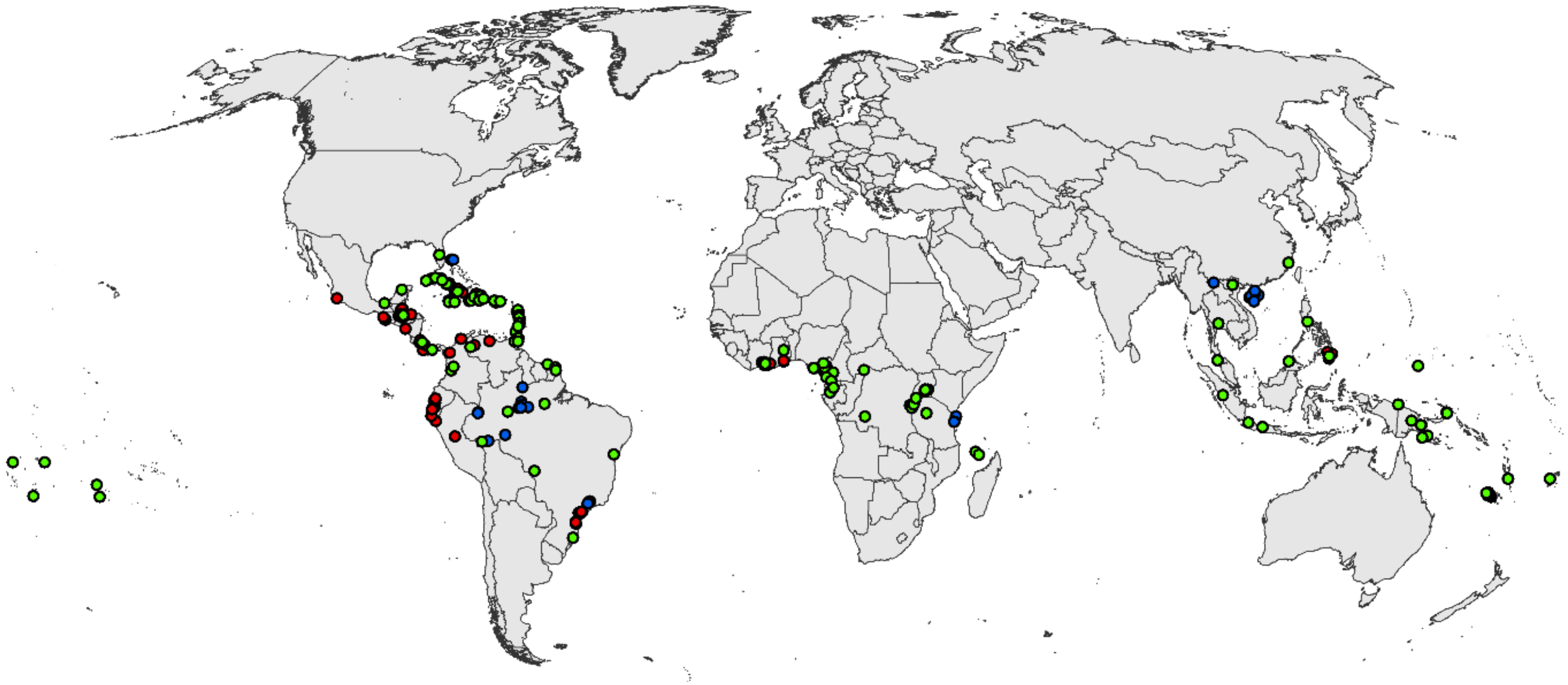

Supplement: S1 Fig — (PDF) [file pone.0220601.s001.pdf]
