# Supplementary material for: Black Sigatoka in bananas: Ecoclimatic suitability and disease pressure assessments
Source: PLoS One. 2019 Aug 14;14(8):e0220601. doi: 10.1371/journal.pone.0220601 (PMC6693783; doi:10.1371/journal.pone.0220601)
Supplement: S2 Fig — (PDF) [file pone.0220601.s002.pdf]

Fig S2. Modelled climate suitability under a natural rainfall scenario of Oceania and Southeast Asia for *P. fijiensis*.

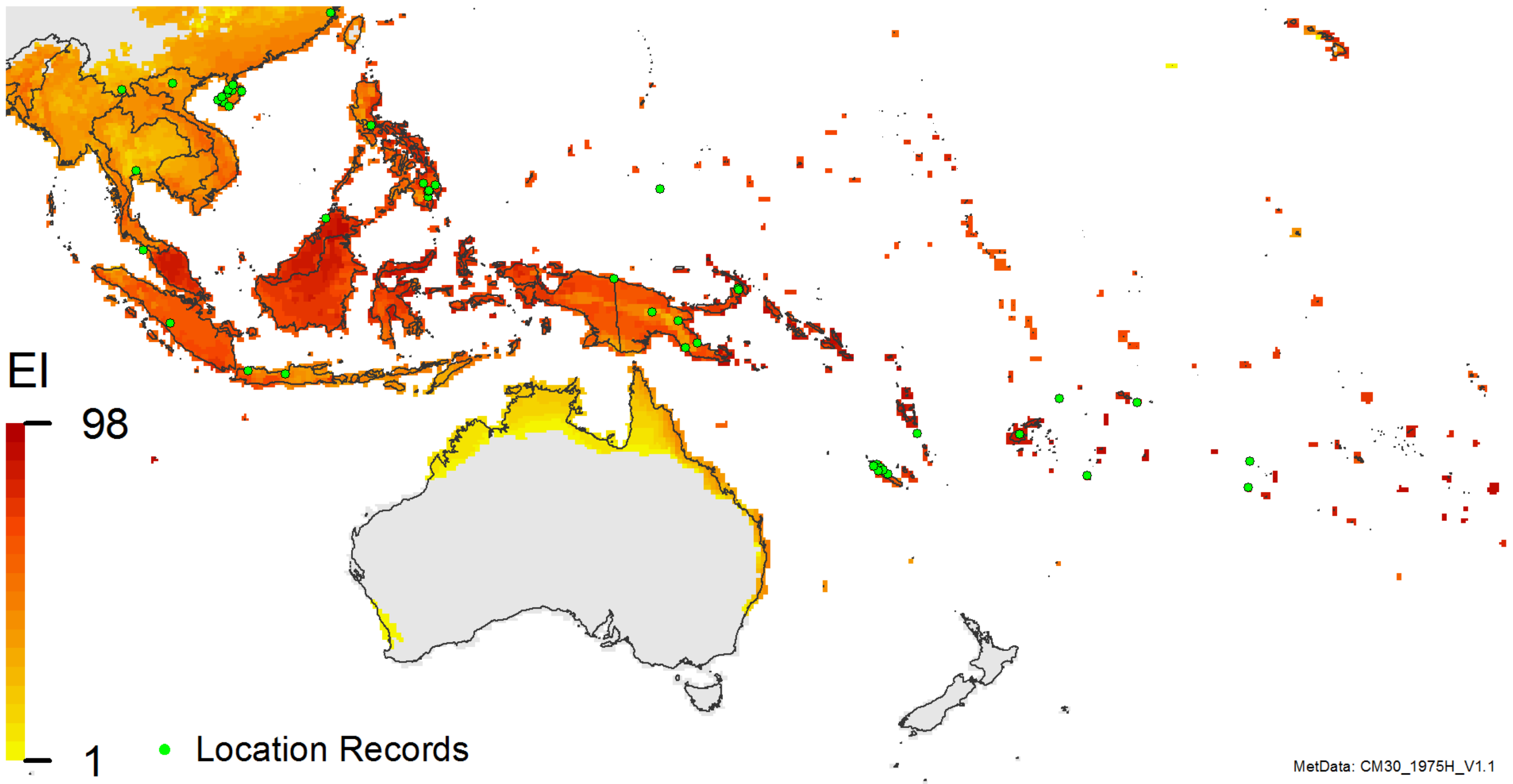

This figure was generated using the parameter values given in Table 1 of the paper, but using the CM30_1975H V1.1 CliMond climatic dataset [1]. This dataset comprises the same 30-year averages of monthly values for daily minimum and maximum temperature (°C), relative humidity (%) at 09:00 and 15:00, and monthly rainfall total (mm), but on a coarser spatial scale, and includes more island land masses. As a result, this map shows that the islands where *P. fijiensis* has been collected are in fact projected to be climatically suitable by our model (the CM10_1975H V1.1 CliMond climatic dataset [1] used for the rest of the paper does not show all of these islands).

1. Kriticos DJ, Webber BL, Leriche A, Ota N, Macadam I, Bathols J, et al. CliMond: global high-resolution historical and future scenario climate surfaces for bioclimatic modelling. Methods Ecol Evol. 2012;3(1):53-64.
