# Supplementary material for: Black Sigatoka in bananas: Ecoclimatic suitability and disease pressure assessments
Source: PLoS One. 2019 Aug 14;14(8):e0220601. doi: 10.1371/journal.pone.0220601 (PMC6693783; doi:10.1371/journal.pone.0220601)
Supplement: S3 Fig — (PDF) [file pone.0220601.s003.pdf]

Fig S3. Growth charts of *P. fijiensis* for two grid cells in the area given by Chuang and Jeger (1) in southern Taiwan. The CLIMEX model was run under a natural rainfall scenario.

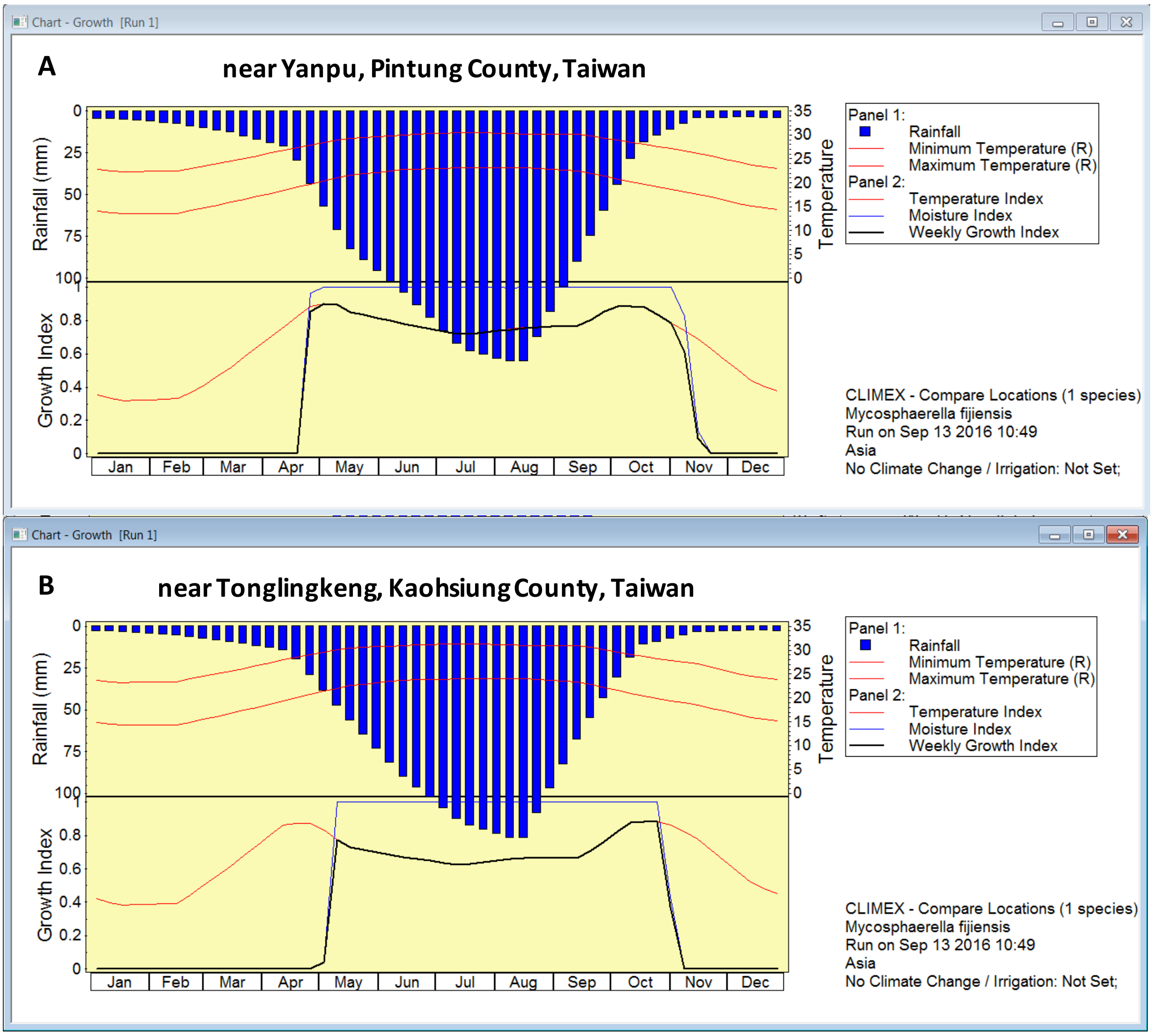

According to Chuang and Jeger (1), *P. fijiensis* first appears in early May in southern Taiwan, and continues throughout the summer, suggesting that the banana plants are available beyond June. In accordance with this, CLIMEX growth charts for this area show that under natural rainfall conditions, *P. fijiensis* can grow from the middle of April until the middle of November, depending on rainfall.

1. Chuang T, Jeger M. Predicting the rate of development of black sigatoka (*Mycosphaerella fijiensis* var. *difformis*) disease in southern Taiwan. Phytopathology. 1987;77(11):1542-7.
