# Supplementary material for: Black Sigatoka in bananas: Ecoclimatic suitability and disease pressure assessments
Source: PLoS One. 2019 Aug 14;14(8):e0220601. doi: 10.1371/journal.pone.0220601 (PMC6693783; doi:10.1371/journal.pone.0220601)
Supplement: S4 Fig — (PDF) [file pone.0220601.s004.pdf]

Fig S4. Growth charts for two locations in Fiji. The CLIMEX model was run under a natural rainfall scenario. (A) is in the centre of the main island, with the lowest EI value in Fiji (EI = 70). (B) is in the centre of the Northern Division, with an EI value of 88.

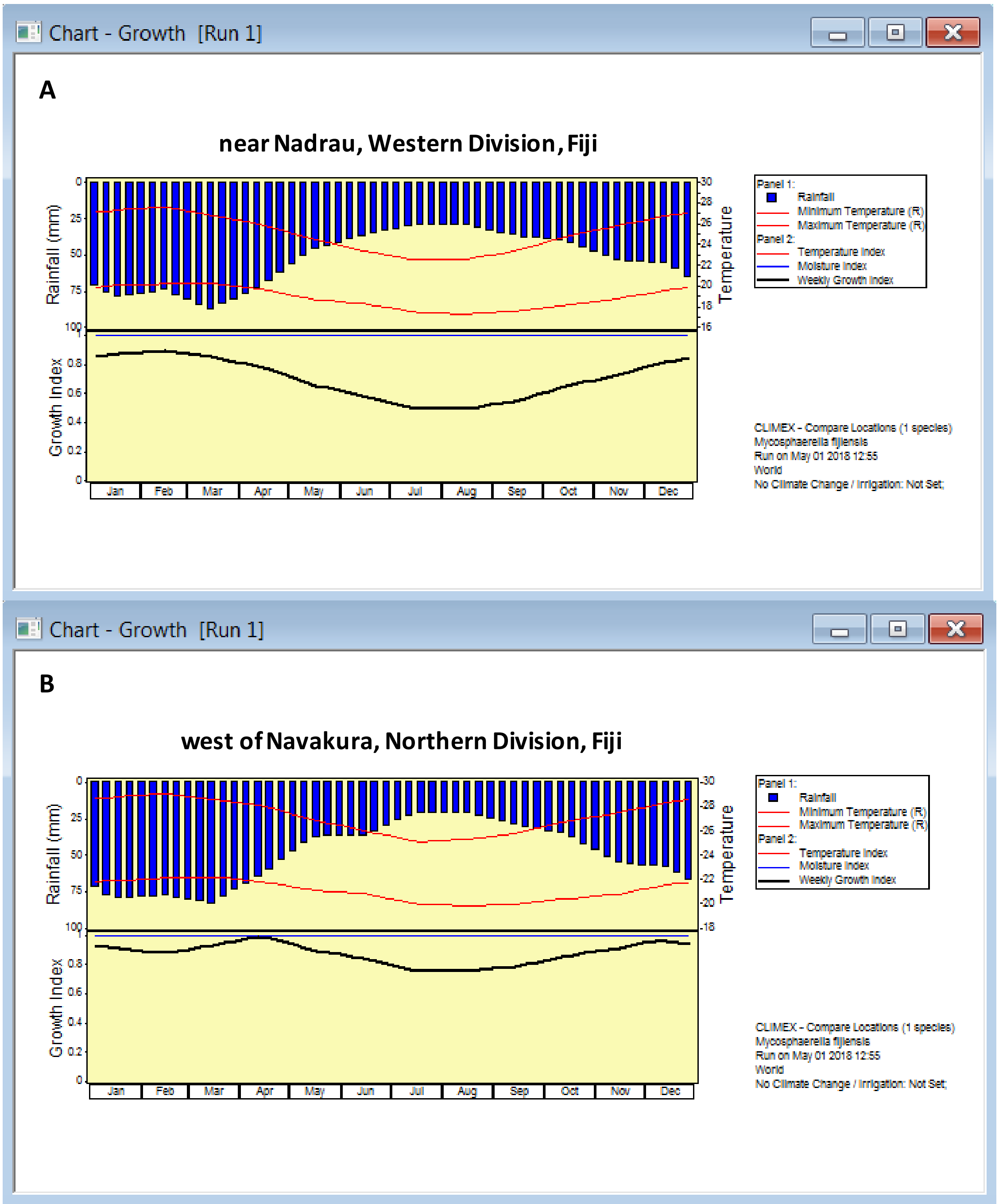

Wardlaw (1) indicates that in Fiji, the pathogen is relatively inactive in the cool period of July to November (p675); that slightly different symptoms in this period are likely due to slower growth (p681-684); and that in mid-June, when night temperatures are below 21 °C, it is possible to have a three-week spraying cycle instead of a two-week cycle (p685). Accordingly, CLIMEX growth charts for Fiji show that the growth of *P. fijiensis* is slower in the cooler months of July and August.

1. Wardlaw CW. Banana Diseases Including Plantains and Bananas, Second Edition. London, UK: Longman Group Limited; 1972. 878 p.
