# Supplementary material for: Black Sigatoka in bananas: Ecoclimatic suitability and disease pressure assessments
Source: PLoS One. 2019 Aug 14;14(8):e0220601. doi: 10.1371/journal.pone.0220601 (PMC6693783; doi:10.1371/journal.pone.0220601)
Supplement: S5 Fig — (PDF) [file pone.0220601.s005.pdf]

Fig S5. Composite map of the modelled climate suitability of Australia for *P. fijiensis*, based on identified irrigation areas [1]. (A) Composite of natural rainfall and 5 mm day^{-1} top-up irrigation; (B) composite of natural rainfall and 7 mm day^{-1} top-up irrigation.. 1 = Babinda; 2 = Tully; 3 = Cardwell; 4 = Bundaberg; 5 = Kempsey; 6 = Carnarvon; 7 = Kununurra.

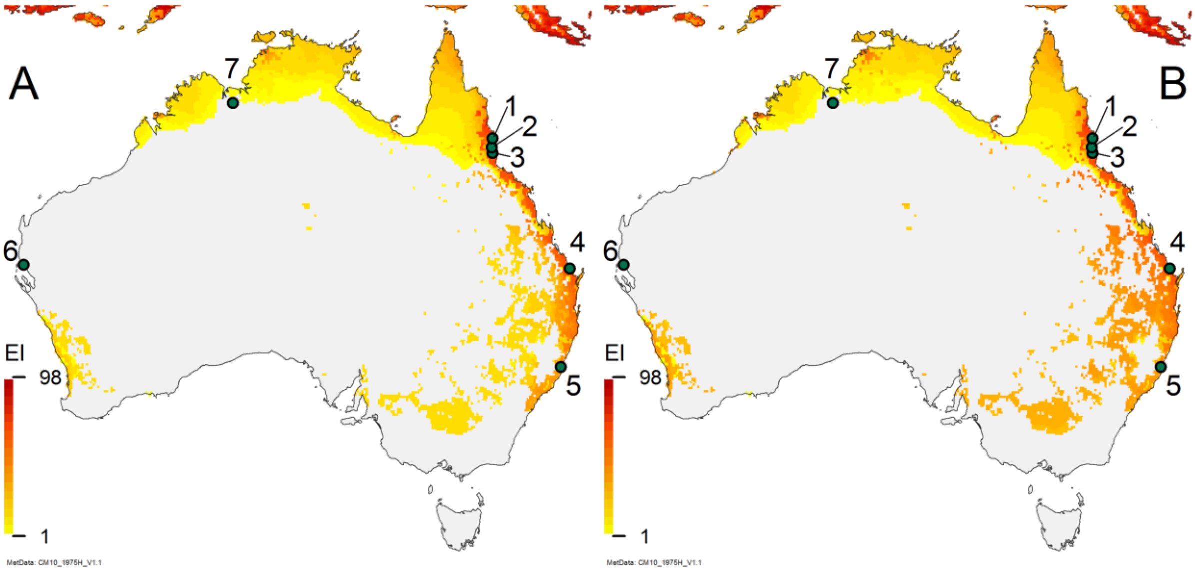

Banana production in Australia generally requires irrigation [2, 3]. Increased irrigation enhances the projected suitability of many regions of Australia. Neither Carnarvon (6) nor Kunannurra (7) are suitable for the establishment and persistence of *P. fijiensis* under a natural rainfall scenario (Fig 2a, Fig S2): Kununurra because of excessive Heat Stress, Carnarvon because of excessive Dry Stress. At Kununurra (7), a 7 mm day^{-1} top-up irrigation scenario is necessary to allow growth during the cooler months (see also Fig S6b). At Carnarvon (6), the 5 mm day^{-1} top up irrigation scenario is sufficient to remove the restrictions of Dry Stress and enable growth to occur in all but the driest couple of months, and the 7 mm day^{-1} top up irrigation scenario enables growth throughout the year (Fig S7). Unfortunately, it would appear that this area is not yet indicated as an irrigated agricultural area [1], and so does not show up as being suitable in these maps (Fig 2b, Fig S5).

1. Siebert S, Henrich V, Frenken K, Burke J, cartographers. Global Map of Irrigation Areas version 5: Rheinische Friedrich-Wilhelms-University, Bonn, Germany / Food and Agriculture Organization of the United Nations, Rome, Italy; 2013.
2. Biosecurity. Final Import Risk Analysis Report for the Importation of Cavendish Bananas from the Philippines, Part C. Canberra, Australia: Biosecurity Australia, Australia 2008.
3. Anon. The Biology of *Musa* L. (banana). Australia: Department of Health and Ageing, 2008 2008. Report No.
