# Supplementary material for: Black Sigatoka in bananas: Ecoclimatic suitability and disease pressure assessments
Source: PLoS One. 2019 Aug 14;14(8):e0220601. doi: 10.1371/journal.pone.0220601 (PMC6693783; doi:10.1371/journal.pone.0220601)
Supplement: S6 Fig — (PDF) [file pone.0220601.s006.pdf]

Fig S6. Growth chart for *P. fijiensis* in Kununurra, Western Australia. (A) with 5 mm day^{-1} and (B) with 7 mm day^{-1} irrigation added as top-up.

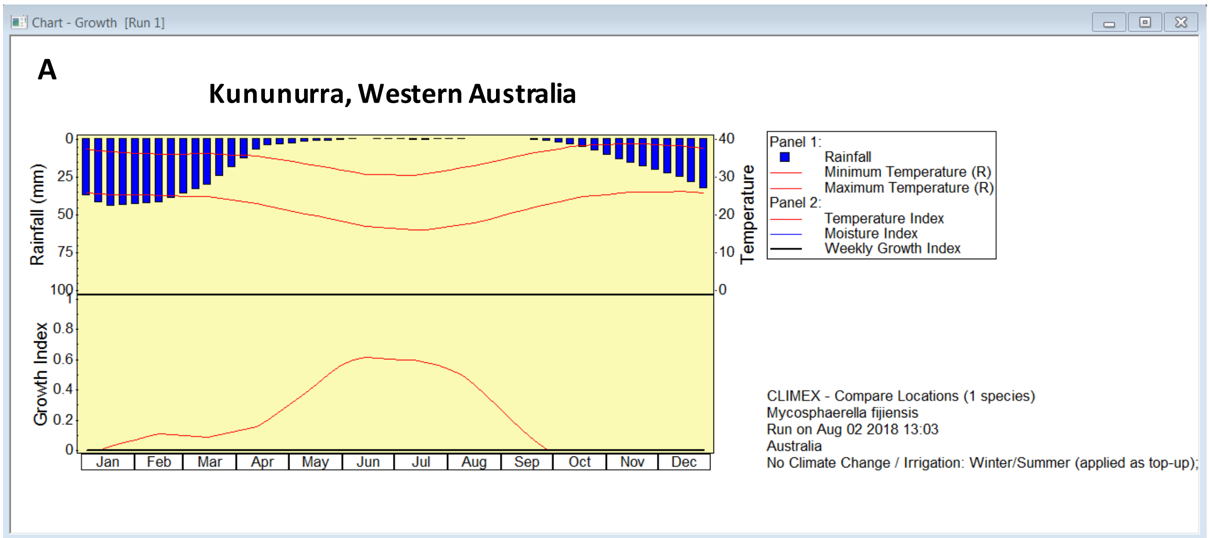

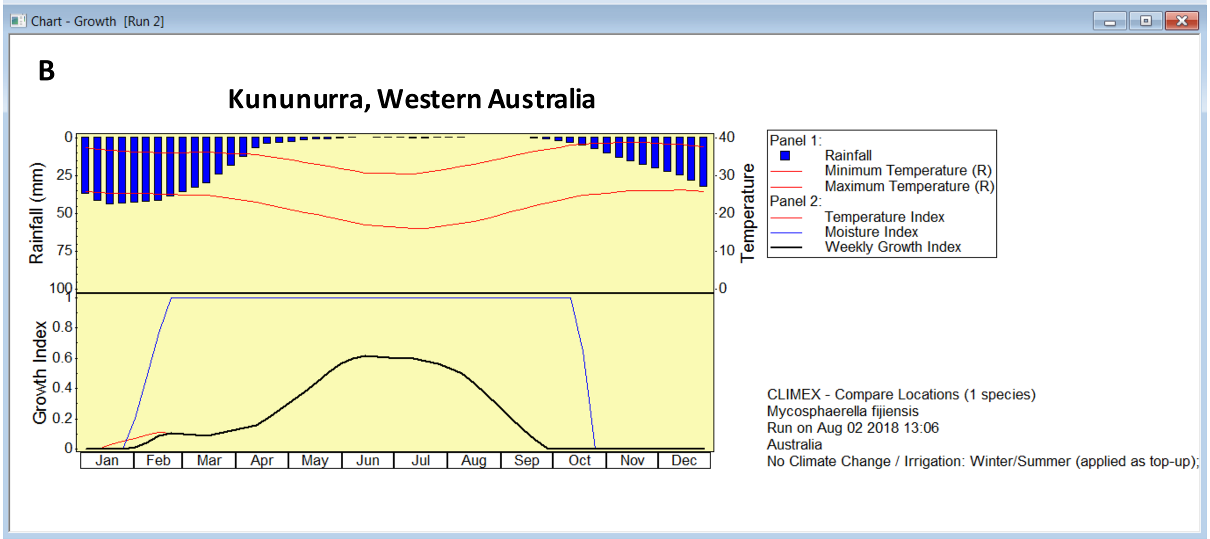

Some banana is grown in Kununurra, Western Australia, [1]. This location experiences excessive Heat Stress (HS = 126) and *P. fijiensis* is not able to persist (EI = 0). An irrigation scenario of 5 mm day^{-1} applied as top-up is still insufficient to allow growth (Fig. S6a): irrigation must be increased to 7 mm day^{-1} (applied as top-up) for growth to occur in the cooler months of February to September (Fig. S6b).

1. Biosecurity Australia. Final Import Risk Analysis Report for the Importation of Cavendish Bananas from the Philippines, Part C. Canberra, Australia: Biosecurity Australia, Australia 2008.
