# Supplementary material for: Black Sigatoka in bananas: Ecoclimatic suitability and disease pressure assessments
Source: PLoS One. 2019 Aug 14;14(8):e0220601. doi: 10.1371/journal.pone.0220601 (PMC6693783; doi:10.1371/journal.pone.0220601)
Supplement: S7 Fig — (PDF) [file pone.0220601.s007.pdf]

Fig S7. Growth charts for *P. fijiensis* in Carnarvon, Western Australia. (A) 5mm day^{-1} irrigation scenario, (B) 7mm day^{-1} irrigation scenario.

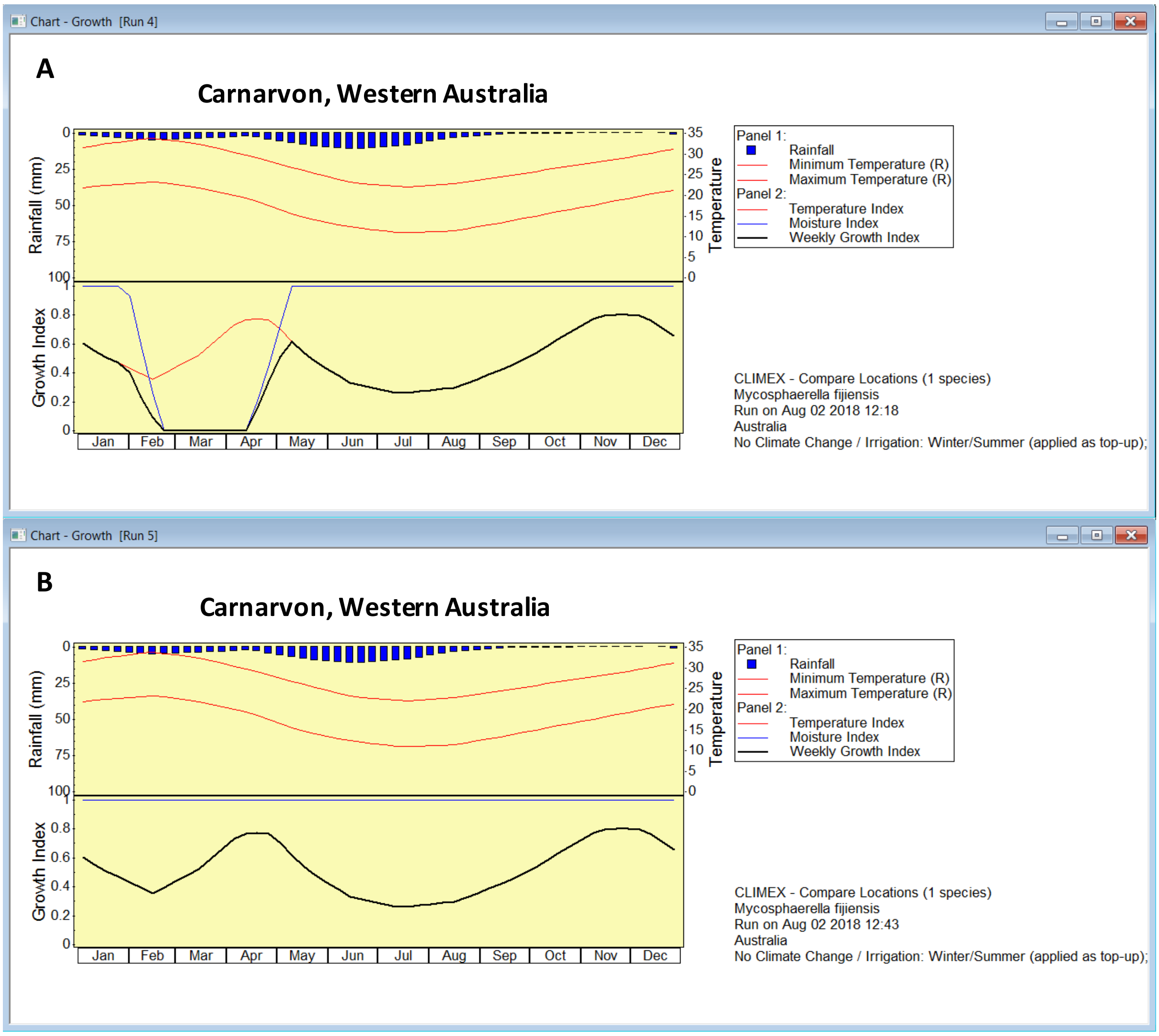

In Carnarvon, excessive Dry Stress (DS = 113) prevents establishment and persistence of *P. fijiensis* under a natural rainfall scenario (Fig 2a, Fig S2). The 5 mm day^{-1} top up irrigation scenario is sufficient to remove this stress and enable growth to occur in all but the driest couple of months (Fig S7A), and the 7 mm day^{-1} top up irrigation scenario enables growth throughout the year (Fig S7B). Unfortunately, it would appear that this area is not yet indicated as an irrigated agricultural area [1], and so does not show up as being suitable in these maps (Fig 2b, Fig S5).

1. Siebert S, Henrich V, Frenken K, Burke J, cartographers. Global Map of Irrigation Areas version 5: Rheinische Friedrich-Wilhelms-University, Bonn, Germany / Food and Agriculture Organization of the United Nations, Rome, Italy; 2013.
