# Supplementary material for: Black Sigatoka in bananas: Ecoclimatic suitability and disease pressure assessments
Source: PLoS One. 2019 Aug 14;14(8):e0220601. doi: 10.1371/journal.pone.0220601 (PMC6693783; doi:10.1371/journal.pone.0220601)
Supplement: S8 Fig — (PDF) [file pone.0220601.s008.pdf]

Fig S8. Modelled climate suitability under a natural rainfall scenario of Central America and the Caribbean for *P. fijiensis*.

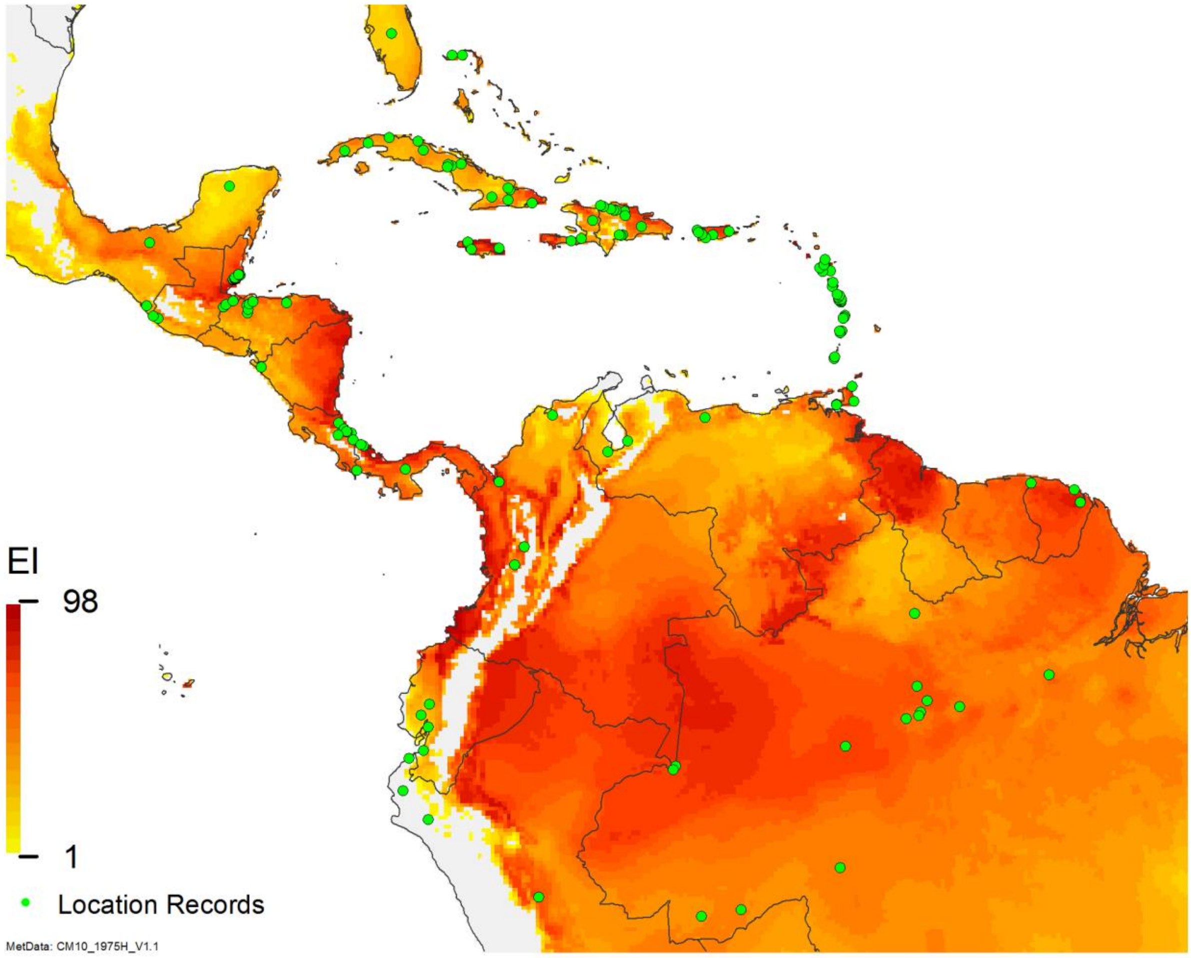

Banana production is rainfed in Costa Rica [1, 2] and Panama [2]. All location records for *P. fijiensis* in Costa Rica fall within highly suitable (EI > 70) regions; those in Panama vary more (EI ranges from 54 to 80), although they are still all highly suitable. Although most of the rest of Central America is projected to be suitable under a natural rainfall scenario, it appears that irrigation is practiced, and so EI values for these locations increase and growth becomes temperature-limited (see Figs. S9-11).

1. Sikirica N. Water Footprint Assessment. Bananas and Pineapples. The Netherlands: Soil and More International, 2011 2011. Report No.
2. LimnoTech. Water Footprint Assessment. Banana and Lettuce Products Produced by Chiquita. Prepared for World Wildlife fund International & Chiquita Brands International. Ann Arbor, Michigan: 2012.
