# Supplementary material for: Black Sigatoka in bananas: Ecoclimatic suitability and disease pressure assessments
Source: PLoS One. 2019 Aug 14;14(8):e0220601. doi: 10.1371/journal.pone.0220601 (PMC6693783; doi:10.1371/journal.pone.0220601)
Supplement: S9 Fig — (PDF) [file pone.0220601.s009.pdf]

Fig S9. Growth chart for *P. fijiensis* in an area representative of Omagua Farm, Guatemala, and expert database site #72. Model was run with 5mm day^{-1} irrigation added as top-up. Growth is temperature-limited under this irrigation scenario.

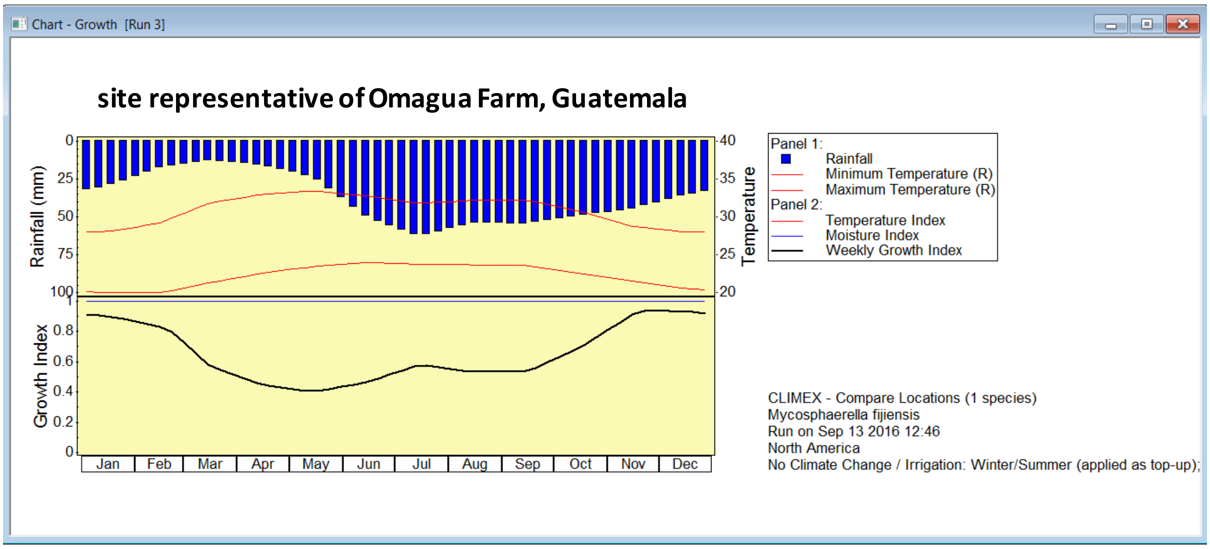

Irrigation of bananas occurs in Guatemala [1-3]. Banana is grown by Chiquita at Omagua Farm in Guatemala [2]. This is near expert database site #72, which falls within CliMond grid cell #158453, graphed above. Under the irrigation scenario of 5 mm day^{-1} added as top-up, EI = 65 and growth of *P. fijiensis* occurs year-round, but with reduced growth in the hotter months.

1. You L, Wood-Sichra U, Fritz S, See L, Koo J. Spatial Production Allocation Model (SPAM) 2005 v2.0 (http://mapspam.info). 2014.
2. LimnoTech. Water Footprint Assessment. Banana and Lettuce Products Produced by Chiquita. Prepared for World Wildlife fund International & Chiquita Brands International. Ann Arbor, Michigan: 2012.
3. FAO. AQUASTAT Website: Food and Agriculture Organization of the United Nations (FAO); 2016 [13 September 2016]. Available from: http://www.fao.org/nr/water/aquastat/countries_regions/.
