# Supplementary material for: Black Sigatoka in bananas: Ecoclimatic suitability and disease pressure assessments
Source: PLoS One. 2019 Aug 14;14(8):e0220601. doi: 10.1371/journal.pone.0220601 (PMC6693783; doi:10.1371/journal.pone.0220601)
Supplement: S10 Fig — (PDF) [file pone.0220601.s010.pdf]

Fig S10. Growth charts for *P. fijiensis* in two areas representative of Omonita Farm, Honduras. (A) expert database site #73 and (B) expert database site #74. Model was run with 5mm day^{-1} irrigation added as top-up. Growth is temperature-limited.

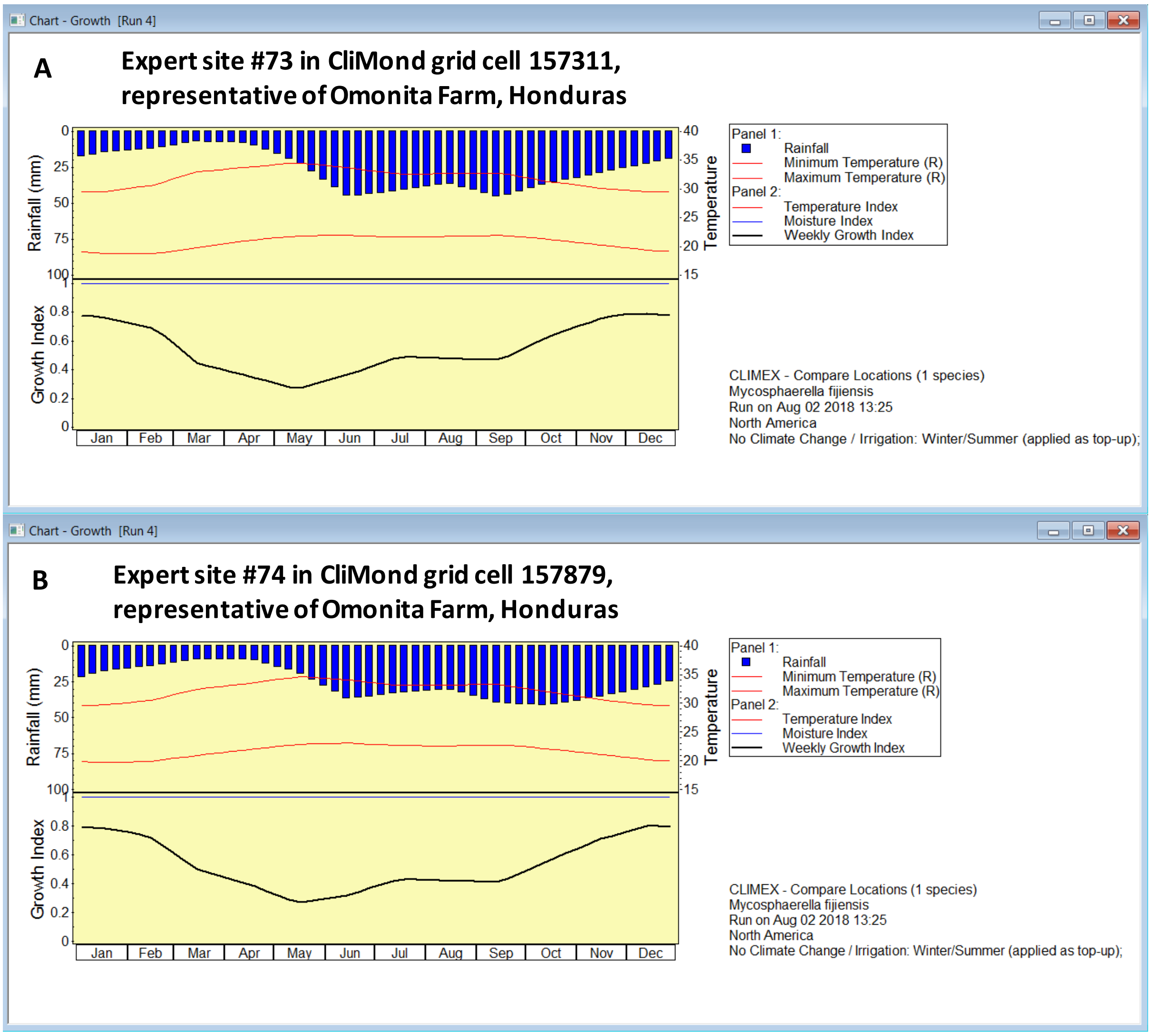

Banana plantations in the Honduras are irrigated [1-3], either all year long [Dole plantations, see p29 2] or from January to August [independent plantations from which Dole purchase bananas, see p31 2]. Omonita Farm in the Honduras [1] is between expert database sites #73 and #74 (CliMond grid cells 157879 and 157311, respectively). Under the irrigation scenario, the EI values are high (53 and 54), with growth occurring year-round, but limited by high temperatures. Not surprisingly, given their proximity to one another, the climate in these two grid cells is very similar.

1. LimnoTech. Water Footprint Assessment. Banana and Lettuce Products Produced by Chiquita. Prepared for World Wildlife fund International & Chiquita Brands International. Ann Arbor, Michigan: 2012.
2. Sikirica N. Water Footprint Assessment. Bananas and Pineapples. The Netherlands: Soil and More International, 2011 2011. Report No.
3. FAO. AQUASTAT Website: Food and Agriculture Organization of the United Nations (FAO); 2016 [13 September 2016]. Available from: http://www.fao.org/nr/water/aquastat/countries_regions/.
