# Supplementary material for: Black Sigatoka in bananas: Ecoclimatic suitability and disease pressure assessments
Source: PLoS One. 2019 Aug 14;14(8):e0220601. doi: 10.1371/journal.pone.0220601 (PMC6693783; doi:10.1371/journal.pone.0220601)
Supplement: S11 Fig — (PDF) [file pone.0220601.s011.pdf]

Fig S11. Growth chart for *P. fijiensis* at expert database site #80 in Nicaragua. Model was run with 5mm day^{-1} irrigation added as top-up.

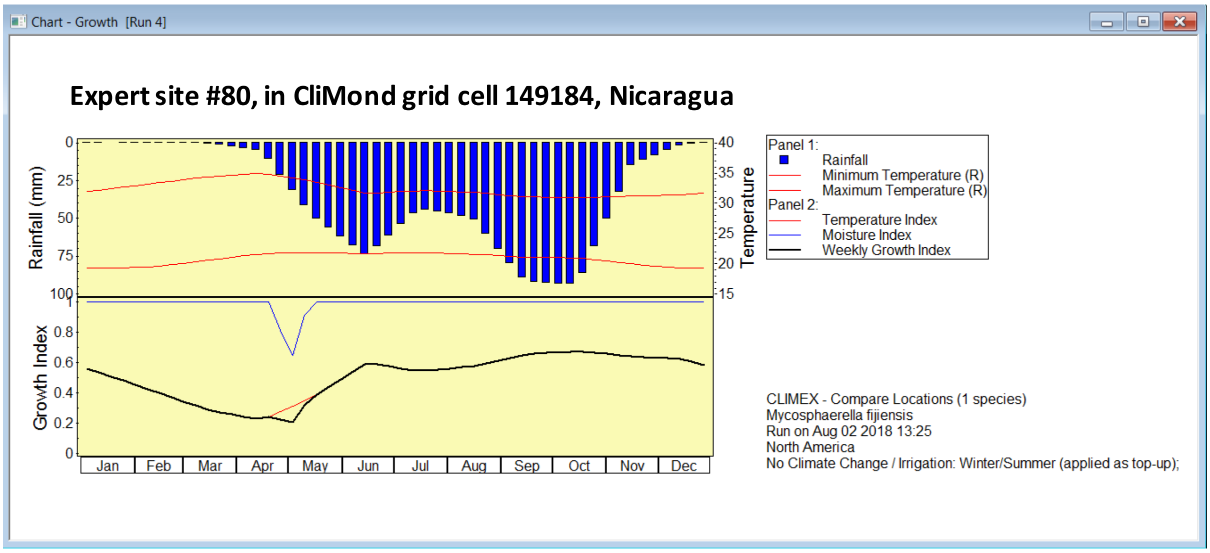

Bananas and plantains are amongst the main crops grown with irrigation in Nicaragua [1]. Growth of *P. fijiensis* at this expert database site is primarily temperature-limited, with only a few weeks of moisture restriction on growth.

1. FAO. AQUASTAT Website: Food and Agriculture Organization of the United Nations (FAO); 2016 [13 September 2016]. Available from: http://www.fao.org/nr/water/aquastat/countries_regions/.
