# Supplementary material for: Black Sigatoka in bananas: Ecoclimatic suitability and disease pressure assessments
Source: PLoS One. 2019 Aug 14;14(8):e0220601. doi: 10.1371/journal.pone.0220601 (PMC6693783; doi:10.1371/journal.pone.0220601)
Supplement: S12 Fig — (PDF) [file pone.0220601.s012.pdf]

Fig S12. Modelled climate suitability of South America for *P. fijiensis.* (A) under natural rainfall scenario and (B) the composite of natural rainfall and 5 mm day^{-1} top-up irrigation, based on identified irrigation areas [1].

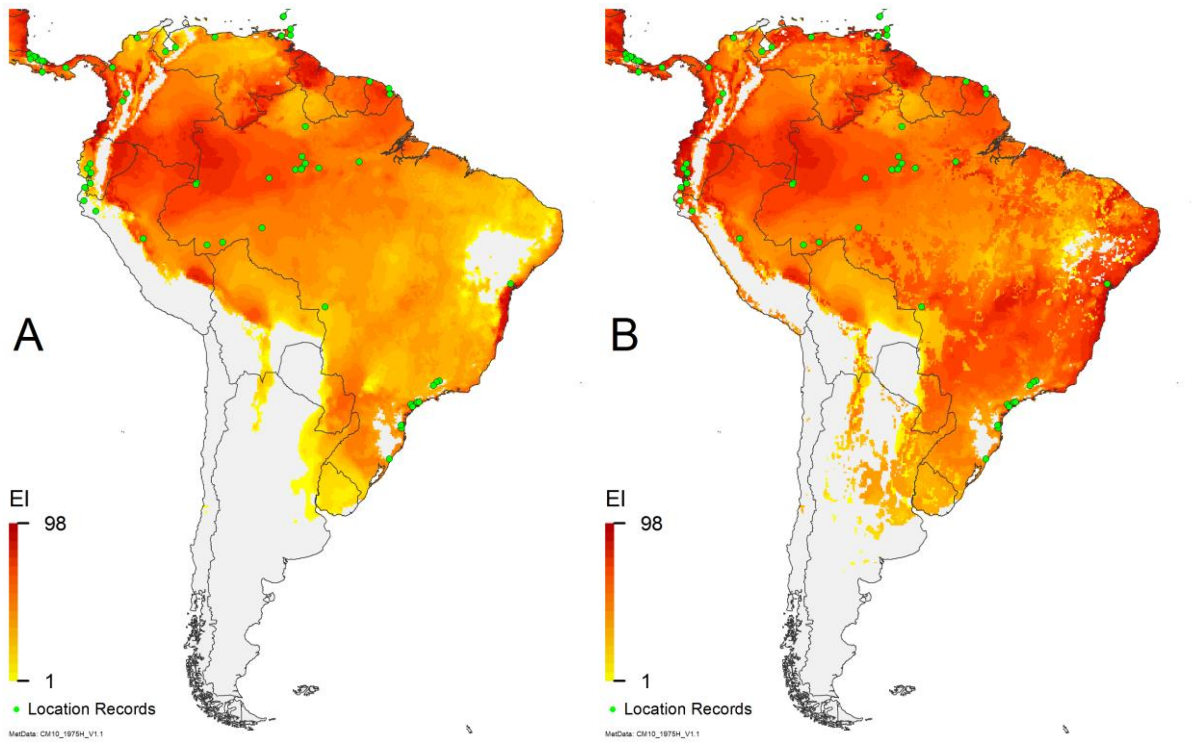

A blog by Andy Jarvis in 2011 (http://dapa.ciat.cgiar.org/desert-bananas/) indicates that the desert region of north-western Peru (Piura) has a booming organic banana business, as a result of flooding their fields from the Chira and Piura Rivers. According to this blog, black Sigatoka does not occur here. However, in 2012, low levels of BLSD were reported in both the Tubes and Piura regions of Peru (https://www.freshfruitportal.com/news/2012/03/02/the-peruvian-organic-banana-advantage/), and the three location records from the expert database clearly indicate that *P. fijiensis* is present. This supports the CLIMEX model, as it shows that P. *fijiensis* can only occur here with the addition of irrigation.

1. Siebert S, Henrich V, Frenken K, Burke J, cartographers. Global Map of Irrigation Areas version 5: Rheinische Friedrich-Wilhelms-University, Bonn, Germany / Food and Agriculture Organization of the United Nations, Rome, Italy; 2013.
