# Supplementary material for: Black Sigatoka in bananas: Ecoclimatic suitability and disease pressure assessments
Source: PLoS One. 2019 Aug 14;14(8):e0220601. doi: 10.1371/journal.pone.0220601 (PMC6693783; doi:10.1371/journal.pone.0220601)
Supplement: S13 Fig — (PDF) [file pone.0220601.s013.pdf]

Fig S13. Modelled climate suitability of the Caribbean for *P. fijiensis*. (A) under natural rainfall scenario and (B) the composite of natural rainfall and 5 mm day^{-1} top-up irrigation, based on identified irrigation areas [1].

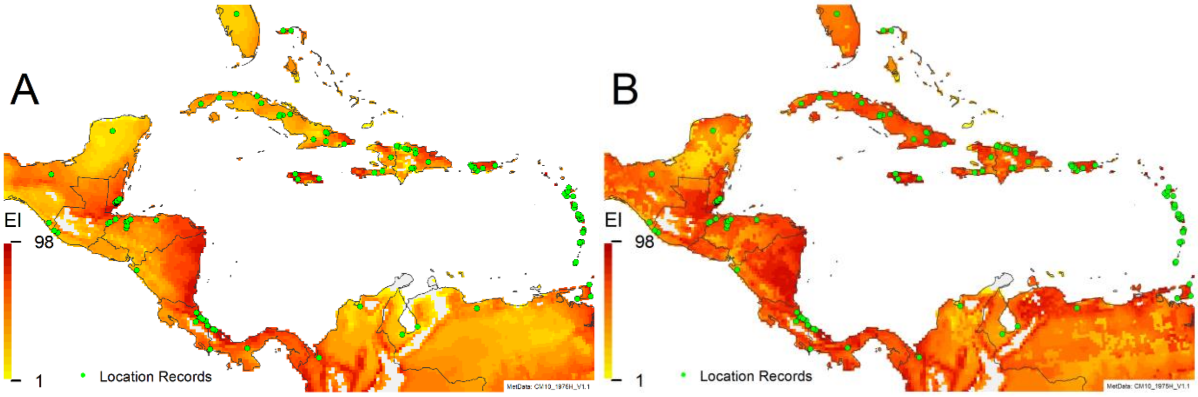

In Haiti, although banana production is traditionally rainfed [2], the more recent organic cultivation of banana in the northeast of Haiti occurs because the dry climate here limits the impact of *P. fijiensis*, and irrigation is necessary for banana production (http://www.fruitrop.com/en/Articles-by-subject/Full-country-profile/2015/Organic-banana-in-Haiti).

In the Dominican Republic, *P. fijiensis* is a problem in high rainfall areas with average temperatures of 27 °C [3]. Banana production in the Dominican Republic is now concentrated in the northwest provinces of Valverde and Monte Christi, and the southern provinces of Azua and Barahona, partially due to the low incidence of BLSD (http://www.fao.org/world-banana-forum/projects/good-practices/organic-production-dominican-republic/en/#.WxCm5JUUmcg). Five locations in the Monte Christi and Valverde provinces are too dry under a natural rainfall scenario (EI = 0), and two other locations in the south (Azua Province) are also very dry (EI = 1). The irrigation scenario makes the moisture index optimal, and vastly increases the EI values in all of these locations (between 47 and 55). Our irrigation scenario compares favourably to the year-round irrigation applied to banana plantations, which varies between the equivalent of 1 mm day^{-1} in Azua in October to 4.7 mm day^{-1} in MonteChristi/Valverde in July (http://goodstuffinternational.com/images/PDF/00---Reporte-final-Evaluacion-Huella-Hidrica-Republica-Dominicana---abril-de-2017.pdf, p24). In Valverde, there is a relatively low disease pressure, and in Azua, yellow Sigatoka disease is more predominant than BLSD (CA, pers. obs.). Only five grid cells in the high altitude (> 1 440 m), mountainous region of the central Dominican Republic remain unsuitable, with insufficient degree-days to complete a generation.

1. Siebert S, Henrich V, Frenken K, Burke J, cartographers. Global Map of Irrigation Areas version 5: Rheinische Friedrich-Wilhelms-University, Bonn, Germany / Food and Agriculture Organization of the United Nations, Rome, Italy; 2013.
2. Ludger J-S. Evaluation of banana and plantain (*Musa* spp.) cultivars in the south of Haiti. Proc Fl St Hortic Soc. 2005;118:258-9.
3. Anon. Manejo integrado de la sigatoka negra en la República Dominicana [14 October 2016]. Available from: http://www.cnmsf.gob.do/Portals/0/docs/Manejo%20Integrado%20de%20la%20Sigatoka%20negra.pdf.
