# Supplementary material for: Black Sigatoka in bananas: Ecoclimatic suitability and disease pressure assessments
Source: PLoS One. 2019 Aug 14;14(8):e0220601. doi: 10.1371/journal.pone.0220601 (PMC6693783; doi:10.1371/journal.pone.0220601)
Supplement: S14 Fig — (PDF) [file pone.0220601.s014.pdf]

Fig S14. Modelled climate suitability of Africa for *P. fijiensis*. (A) under natural rainfall scenario and (B) the composite of natural rainfall and 5 mm day^{-1} top-up irrigation, based on identified irrigation areas [1].

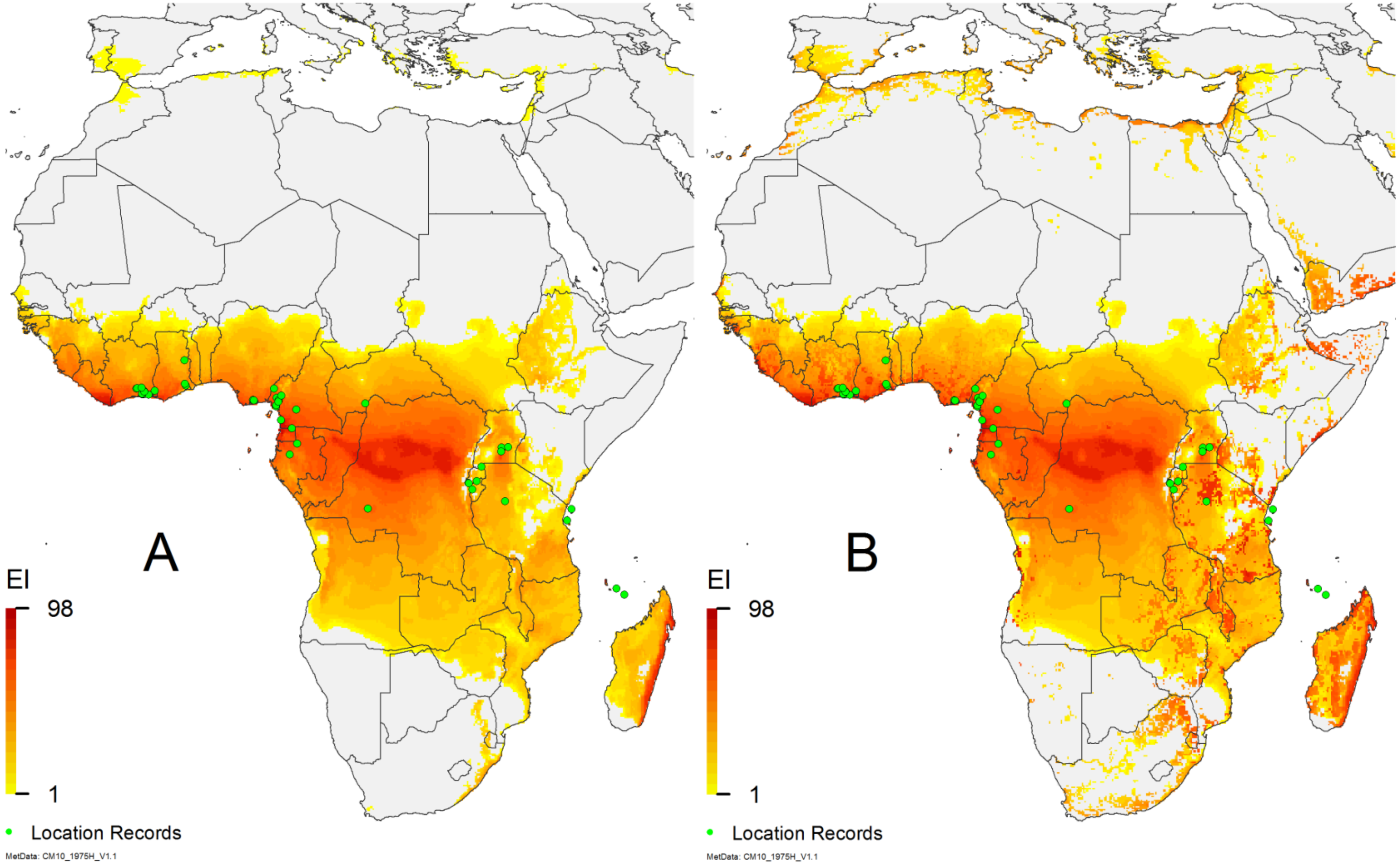

1. Siebert S, Henrich V, Frenken K, Burke J, cartographers. Global Map of Irrigation Areas version 5: Rheinische Friedrich-Wilhelms-University, Bonn, Germany / Food and Agriculture Organization of the United Nations, Rome, Italy; 2013.
