# Supplementary material for: Black Sigatoka in bananas: Ecoclimatic suitability and disease pressure assessments
Source: PLoS One. 2019 Aug 14;14(8):e0220601. doi: 10.1371/journal.pone.0220601 (PMC6693783; doi:10.1371/journal.pone.0220601)

Fig S16. Soil moisture values of the CLIMEX grid cells containing the ten highest-ranked locations (lines with markers) and the five lowest-ranked locations (solid lines, no markers) of *P. fijiensis*. For each location, grid cell number, country, disease pressure ranking, and EI value without irrigation (and with irrigation in brackets) are given.

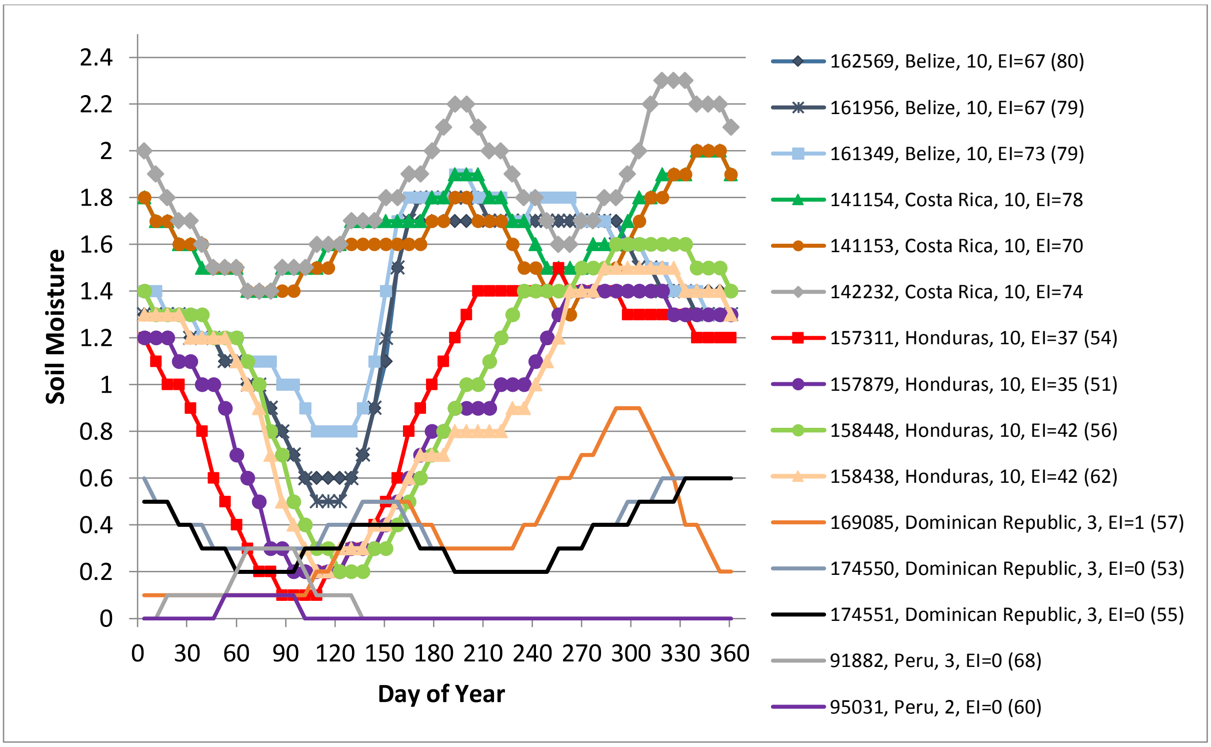

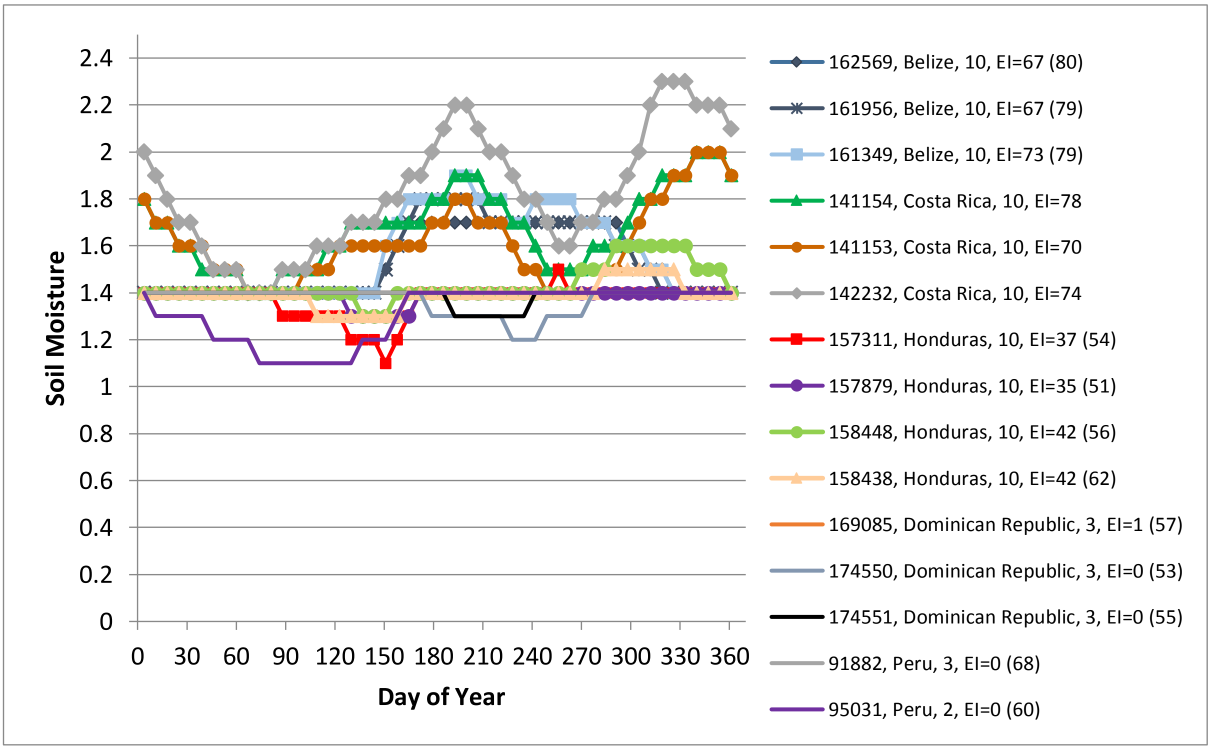

Supplement: S16 Fig — (PDF) [file pone.0220601.s016.pdf]
