# Supplementary material for: Black Sigatoka in bananas: Ecoclimatic suitability and disease pressure assessments
Source: PLoS One. 2019 Aug 14;14(8):e0220601. doi: 10.1371/journal.pone.0220601 (PMC6693783; doi:10.1371/journal.pone.0220601)
Supplement: S1 Table — (PDF) [file pone.0220601.s017.pdf]

Table S1. Water requirements for optimal banana production.

**Source**	**Weekly water requirement (mm)**	**Annual rainfall for rainfed production (mm)**
Anon. (1)	50 – 100	
Diczbalis (2), for Darwin and Katherine, Australia	39 – 66	
https://www.daf.qld.gov.au/plants/fruit-and-vegetables/fruit-and-nuts/bananas/frequently-asked-questions-about-bananas/irrigating	20 – 60	
http://www.fao.org/nr/water/cropinfo_banana.html	[equivalent to 38 – 48]	2 000 – 2 500
http://www.memoireonline.com/02/11/4268/Design-of-a-geographic-information-supported-database-for-the-management-of-pressurised-irrigation-s.html	30-40	1 500-2 000
https://www.agric.wa.gov.au/bananas/banana-irrigation-and-soil-water-management-oria	growers applied 55 - 85 mm H_2O / hectare / week in summer, and 30 - 60mm in winter. The range in annual application of irrigation water in the ORIA is about 17 to 26ML/ha.	

1. Anon. The Biology of *Musa* L. (banana). Australia: Department of Health and Ageing, 2008 2008. Report No.
2. Diczbalis Y. Irrigation management of bananas in the Top End1993 20 September 2016:[1-7 pp.]. Available from: https://dpif.nt.gov.au/__data/assets/pdf_file/0009/233766/567.pdf.
